# Single molecule localisation microscopy reveals how HIV-1 Gag proteins sense membrane virus assembly sites in living host CD4 T cells

**DOI:** 10.1038/s41598-018-34536-y

**Published:** 2018-11-02

**Authors:** Charlotte Floderer, Jean-Baptiste Masson, Elise Boilley, Sonia Georgeault, Peggy Merida, Mohamed El Beheiry, Maxime Dahan, Philippe Roingeard, Jean-Baptiste Sibarita, Cyril Favard, Delphine Muriaux

**Affiliations:** 1 Infectious Disease Research Institute of Montpellier (IRIM), UMR9004 CNRS, University of Montpellier, 1919 route de Mende, 34293 Montpellier, France; 2 Decision and Bayesian Computation, UMR 3571 CNRS, Pasteur Institute, Paris, France; 3 INSERM U966 and IBiSA EM Facility, University of Tours, Tours, France; 4 Light and Optical Control of Cellular Organization, Curie Institute, UMR, 168 CNRS Paris, France; 5 Interdisciplinary Institute for Neuroscience, UMR 5297 CNRS, University of Bordeaux, Bordeaux, France

## Abstract

Monitoring virus assembly at the nanoscale in host cells remains a major challenge. Human immunodeficiency virus type 1 (HIV-1) components are addressed to the plasma membrane where they assemble to form spherical particles of 100 nm in diameter. Interestingly, HIV-1 Gag protein expression alone is sufficient to produce virus-like particles (VLPs) that resemble the immature virus. Here, we monitored VLP formation at the plasma membrane of host CD4^+^ T cells using a newly developed workflow allowing the analysis of long duration recordings of single-molecule Gag protein localisation and movement. Comparison of Gag assembling platforms in CD4^+^ T cells expressing wild type or assembly-defective Gag mutant proteins showed that VLP formation lasts roughly 15 minutes with an assembly time of 5 minutes. Trapping energy maps, built from membrane associated Gag protein movements, showed that one third of the assembling energy is due to direct Gag capsid-capsid interaction while the remaining two thirds require the nucleocapsid-RNA interactions. Finally, we show that the viral RNA genome does not increase the attraction of Gag at the membrane towards the assembling site but rather acts as a spatiotemporal coordinator of the membrane assembly process.

## Introduction

Enveloped RNA viruses are small entities that bud from the host cell plasma membrane. The formation of these nanoscopic assemblies requires hundreds of viral proteins to oligomerise at the inner face of the cell membrane before budding. How are single viral proteins recruited to virus budding sites once at the cell plasma membrane? What are the relative contributions of viral protein-protein, or protein-RNA genome interactions to this membrane recruitment? A method to decipher the underlying dynamic molecular mechanisms of virus assemblies at cell membranes, molecule by molecule, is super-resolution microscopy applied to living cells. This requires the combination of tools to enable the nanoscale analysis of viral proteins dynamics at high densities over long periods of time. For instance, recent progress in single-molecule localisation microscopy allows deciphering protein organisation and dynamics in a single cell at the nanoscale level^1–3^. In this context, we studied HIV-1 assembly and budding at the plasma membrane of living host CD4^+^ T cells by tracking the viral membrane Gag proteins and its derivatives. Human immunodeficiency virus type 1 (HIV-1) produces particles with a diameter of 100–130 nm filled with approximately 2000 viral Gag proteins. The Gag polyprotein is the main determinant for HIV-1 particle assembly that occurs mainly at the plasma membrane of the host cell^4^. When expressed alone in a cell, HIV-1 Gag proteins can produce non-infectious virus-like particles (VLPs) that resemble immature viruses, but do not require maturation (encoded by the Pol gene) or envelope proteins (encoded by the Env gene). Therefore, it is a powerful tool for studying virus assembly mechanisms in a minimally productive system^5^. The HIV-1 Gag polyprotein is made of the following domains: Matrix protein p17 (MA), Capsid protein p24 (CA), Nucleocapsid protein p7 (NC) as well as the p6 domain and two spacer peptides (sp1 and sp2). MA is myristoylated and contains a highly basic region involved in Gag targeting and anchoring to the inner leaflet of the host cell plasma membrane where viral assembly occurs (reviewed in^6–8^). CA, via CA-CA interacting domains, promote Gag-Gag oligomerisation *in vitro*^9,10^ and in cells^11^. NC, sp2 and p6 are required for Gag assembly and particle budding. Specifically, the nucleocapsid (NC) domain of Gag recruits the genomic RNA, but can also interact with cellular RNAs, to favor Gag-Gag oligomerisation on the RNA template, *in vitro* and in cells (reviewed in^12,13^) and is also involved in virus assembly^13,14^. The p6 domain of Gag recruits the cellular ESCRT proteins required for viral particle release^15,16^. Sp1, at the end of the capsid (CA), acts as a molecular switch for HIV-1 assembly^17^. In this work, we explored how HIV-1 Gag derivatives, mutated or deleted from different domains, affect membrane Gag recruitment into the viral bud during its formation.

The study of HIV-1 Gag assembly at the plasma membrane of living cells was first done by Jouvenet, Ivanchenko and collaborators: kinetics of fluorescent-labelled Gag assembly and VLP formation have been described in adherent HeLa cells by measuring the local increase in fluorescent intensity of single virions^18,19^. In these cells, it was estimated that 5 to 6 minutes were required for Gag VLP assembly in the absence of genomic RNA^18^ and about 20 minutes to complete 90% of Gag VLP assembly and budding^19^. Jouvenet *et al*.^20^ also reported that HIV-1 Gag and a fluorescent tagged pseudo-viral RNA assemble together at the cell plasma membrane. In addition, it was recently shown that HIV-1 Gag assembly at the plasma membrane takes place at sites where the viral RNA is targeted^21^. The viral Gag proteins appears to stabilise the viral RNA at the plasma membrane and between 1/10 to 1/3 of the viral RNA is packaged into nascent particles within 30 minutes^22^. These interactions between the viral genome and Gag seem to enhance virus assembly^23^. However, these studies were never performed in the natural host cells of HIV-1, i.e., CD4^+^ T cells. Moreover, although these results suggest that the viral genomic RNA encoding Gag acts as a catalyser for virus assembly, they do not quantitatively assess the effect of the viral genomic RNA on the spatio-temporal coordination of HIV-1 Gag assembly at the host cell plasma membrane. In this study we succeeded to quantify HIV-1 single Gag protein dynamics at the nanoscale level during immature virus assembly at the plasma membrane of living host CD4^+^ T cells. We measured the respective roles of different known Gag domains and of the viral RNA genome in the immature VLP formation. To this end, the photoactivatable fluorescent protein mEOS2 was introduced into the HIV-1 Gag precursor (Gag(i)mEOS2) and derivatives. This tag is compatible with live photoactivated localisation microscopy (PALM)^1,3^. Live PALM provides a precise spatiotemporal description of VLP formation at the plasma membrane of individual cells, with a spatial resolution of about 50 nm and a temporal resolution in the millisecond range. By coupling live PALM, TIRF-microscopy, Bayesian inference and hidden Markov statistical analyses based on millions of Gag protein localisations and hundreds of buds, we were able to monitor HIV-1 Gag particle formation at the plasma membrane of host CD4^+^ T cells. Moreover, by comparing wild type (WT) and assembly-defective Gag mutants, we identified which Gag domains are crucial for membrane Gag assembly coordination at the host T cell surface. First, we found that in fixed CD4^+^ T cells, membrane Gag assembly platforms are rarely formed at the T cell surface when the Gag NC-RNA binding domain is removed. Then, based on the temporal changes observed in Gag localisation density maps, we showed that, in CD4^+^ T cells, VLP assembly and budding require between 15 and 20 min. Finally, by combining live PALM and Bayesian inference analysis^24,25^, we extracted from single Gag protein motion an effective trapping energy to decipher membrane Gag recruitment into the bud. We were thus able to quantify, for the first time, Gag trapping energy during particle formation. By analysing the temporal correlation between changes in the density and the trapping energy of membrane Gag molecules during VLP assembly, we brought evidence that the cis-packageable viral genome, which encodes Rev/RRE dependant Gag, spatio-temporally coordinates the complete VLP assembly at the surface of the host CD4^+^ T cells.

## Results

### Expression of WT Gag(i)mEOS2 and assembly-defective mutants in Jurkat T cells

**Figure 1:**
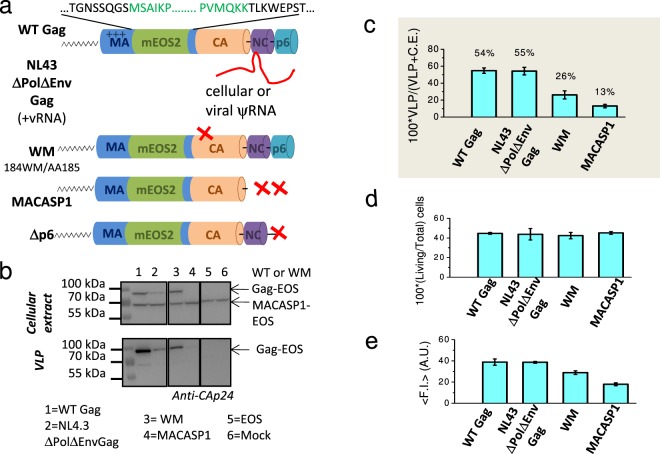
Characterisation of HIV-1 Gag(i)mEOS2 wild type and mutants expressed in Jurkat T cells. (**a**) Schematic representation of HIV-1 WT Gag(i)mEOS2 and derivatives used in this study. (**b**) Western blot analysis of HIV-1 WT Gag(i)mEOS2 and mutant proteins expressed in Jurkat T cells (“cellular extract”), used for live PALM analysis, and the corresponding purified VLPs (“VLP”). Western blots are revealed with an anti-CAp24 antibody. Images of the full-length Western blots are presented in SI (Fig. S1). (**c**) Quantification of WT Gag(i)mEOS2 and mutant VLP production efficiency in transfected Jurkat T cells relative to total Gag protein (mean ± C.I. of 4 to 6 independent experiments). (**d**) Cell viability (relative to the total number of transfected cells) and protein expression of Gag(i)mEOS2 and mutants in Jurkat T cells using flow cytometry (mean ± C.I. of 3 independent experiments, see statistics section in Methods for detailed explanation). (**e**) Global fluorescence intensity (F.I.) of Gag(i)mEOS2 transfected Jurkat T cells as measured by flow cytometry for each condition, as indicated.

First, HIV-1 Gag and assembly defective mutants that harbour the mEOS2 fluorescent protein were generated (Fig. 1a) and characterised for cell expression and VLP production (Fig. 1). The fluorescent protein was introduced between the matrix (MA) and the capsid (CA), thus preserving Gag capacity to assemble and bud from the cell membrane after transient expression in mammalian cells^26^. WT Gag(i)mEOS2 was produced using either the pNL4.3ΔPolΔEnv plasmid that also includes a cis-packaging-signal on the viral RNA that promotes viral RNA genome encapsidation into the nascent VLP (NL4.3ΔPolΔEnv Gag, hereafter)^27^ or the pGag-(i)mEOS2 WT plasmid without this signal (WT Gag, hereafter). The WM, MACASP1, MACASP1/WM and Δp6 Gag mutants were derived from WT Gag(i)mEOS2 (see Methods). WM harbours a mutation in CA that reduces CA-CA interactions and impairs Gag oligomerisation. The MACASP1 mutant carries a stop codon at the end of CA-SP1 and therefore, lacks Gag C-terminus (NC-sp1-p6), thus impairing virus assembly and budding^28^. In Δp6, a deletion in the p6 domain of Gag impairs ESCRT recruitment and consequently particle release^15^. Indeed, tethered Δp6 particles remained attached to the cell membrane (see Fig. S2) as previously reported^15^. All the Gag proteins were well expressed after transient transference in Jurkat CD4^+^ T lymphocytes, as indicated by western blot analysis (Figs 1b and S1) and by flow cytometry (Fig. 1e).

Cell viability analysis by flow cytometry indicated that 40–50% of cells were alive after electroporation (Fig. 1d). Analysis of the geometric mean of fluorescent intensity (Fig. 1e) showed that 24 hours post-transfection, the global protein expression level was comparable for WT Gag and NL4.3ΔPolΔEnvGag, whereas it was 2-fold lower, on average, for MACASP1, WM and mEOS2 (vector alone). Finally, VLP production was assessed by semi-quantitative western blot analysis with an anti-CA antibody (Methods) (Fig. 1b,c). Only purified WT Gag and WM VLPs were easily observed, whereas MACASP1 VLPs were often undetectable (Fig. 1b). In agreement, VLP release calculations showed that VLP production decreased from about 50% to 10% for MACASP1 (Fig. 1c). The capacity of WT Gag and derivatives to bind to cell membranes was verified with membrane flotation assays in HEK293T cells (as described in Thomas *et al*.^28^) in order to know if Gag derivatives will be observable by TIRF microscopy (Fig. S3a). Although Gag was well expressed (Fig. 1b), the fraction of WT Gag bound to cell membranes was between 60 to 80% of total Gag (Fig. S2b), and this value further decreased for WM and MACASP1 (p < 0.01, see statistics section in methods), and even more for the WM/MACASP1 double mutant (p < 0.001). This made analysis of the later impossible by live PALM. These results indicate that upon alteration of Gag multimerisation capacity, Gag is less bound to cell membranes, confirming a role for Gag oligomerisation in stabilising Gag-membrane interactions, in agreement with^29^. Moreover, it was recently shown that *in vitro* Gag oligomerisation occurs also on PIP_2_-containing lipid membranes and that it is reduced by the same WM mutation in the CA domain of Gag^10^. Transmission electron microscopy was then used to check whether WT Gag(i)mEOS2 and mutants formed particles (Gag VLPs) (Fig. S2). Upon expression of WT Gag, cells produced high amounts of electron-dense budding vesicles (i.e., Gag VLPs). After transfection of NL4.3ΔPolΔEnv Gag, particles seemed rarer at the cell surface than in WT Gag-expressing cells. Upon transfection of MACASP1, very rare VLPs were detected, often localised at the plasma membrane, as indicated by the dark staining at the cell membrane (Fig. S2a). HEK293T cells can naturally produce some mock vesicles that are not electron-dense structures (Fig. S2a). However, neither dark staining at the plasma membrane nor VLP was detected in mock cells (Fig. S2a). These results are in agreement with the literature and allowed us to select the HIV-1 Gag protein derivatives that were well expressed in Jurkat T cells and that bound to the cell membrane: two prerequisites for analysing membrane Gag(i)mEOS2 assembly at the T cell surface by live PALM.

### Analyses of membrane Gag and mutant cluster densities in fixed CD4^+^ T cells reveal that the NC domain of Gag is necessary for cluster formation

**Figure 2:**
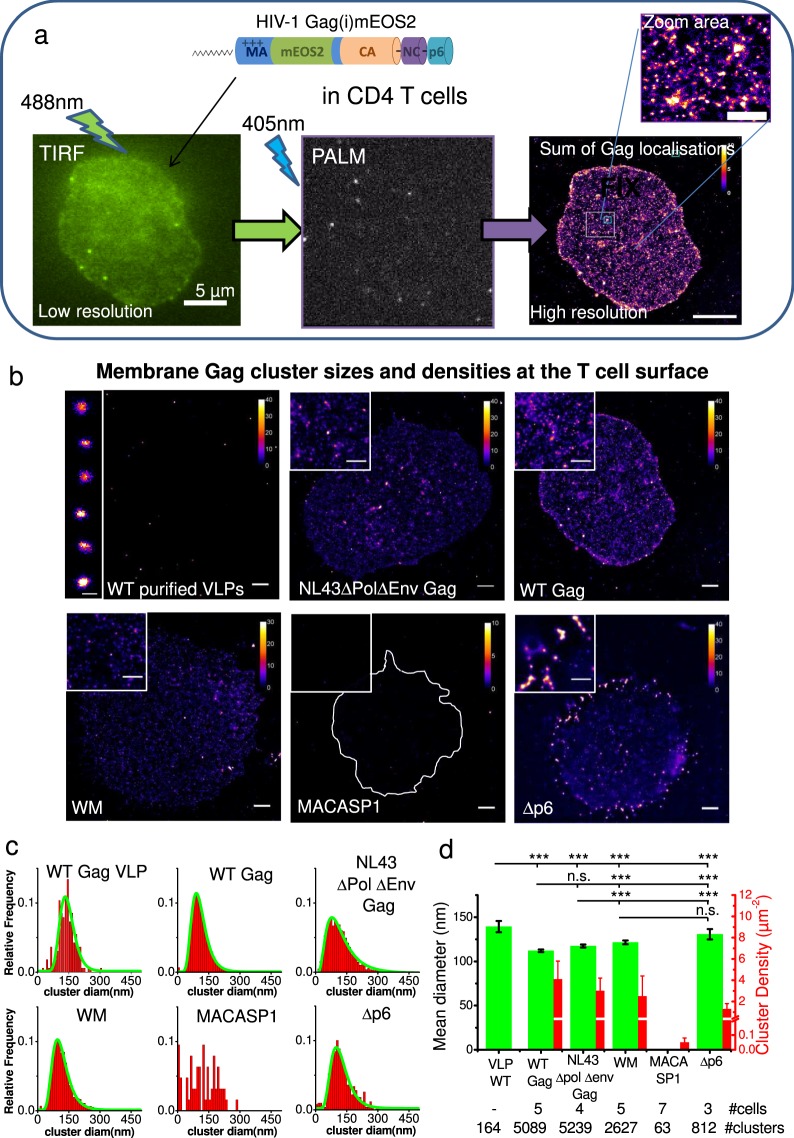
HIV-1 Gag(i)mEOS2 and mutants cluster formation at the surface of Jurkat T cells observed by PALM. (**a**) Low resolution TIRF imaging and high resolution PALM imaging of Jurkat T cells expressing WT Gag(i)mEOS2 that lead after summing all Gag localisations to images of all assembly clusters. (**b**) Jurkat T cells that express WT Gag, NL4.3ΔPolΔEnv Gag, WM, MACASP1 or Δp6 were fixed and PALM imaging of the cell surface was performed using TIRF mode. Purified VLPs (left upper panel) from WT Gag productive cells were also analysed with this method and served as VLP size reference. Each image contains a zoom of a selected area of the depicted cell (2x, upper left inset). Scale bars: 2, 1 *μ*m, respectively. (**c**) Size distribution (red) and their log-normal fit (green, when possible) of the different assembly clusters observed in T-cells. (**d**) In green, plot of the mean diameter (left y-axis) observed for each condition. Error bars are C.I. of the data. Differences observed according to Student’s t-test were performed on log values of the diameter’s distributions. (***p < 1.10^−4^, see Table S1 for exact p values). In red, plot of the assembly clusters surface densities at the cell membrane for each condition, illustrating the drastic decrease for MACASP1. Error bars are C.I. of the surface densities.

We took benefit of single-molecule localisation microscopy to statistically analyse assembly platform sizes and density changes between WT Gag and known assembly defective mutants (Fig. 2). Viral Gag and derivatives formed clusters imaged by PALM in fixed Jurkat T cells (Fig. 2a).

Examples of reconstructed PALM images are presented in Fig. 2b for the two different WT Gag (WT and NL43ΔPolΔEnv) and the two different Gag oligomerisation-defective mutants (WM and MACASP1) as well as for the Gag-ESCRT recruitment defective mutant, Δp6. Figure 2c shows the distributions of the Gag cluster mean diameters for each Gag derivatives and the log-normal fit of the distributions. As shown in Fig. 2d, the diameters of WT Gag (112 ± 2 nm) and NL4.3ΔPolΔEnv Gag (117 ± 2 nm) clusters were similar, but were significantly different to the WM clusters (121 ± 2 nm) (see Table S1 and the statistics section in methods for detailed statistics analysis). This was confirmed by electron microscopy (Fig. S2). Due to the low number of observed MACASP1 clusters, their mean diameter could not be determined. Finally, these mean diameter values were all found to be significantly smaller than the VLP one (139 ± 7 nm) obtained from images of purified VLPs produced by WT Gag-expressing Jurkat T cells (Fig. 2b, WT purified VLPs), suggesting that theses clusters were indeed Gag assembly platforms. The values obtained here are in good agreement with previous PALM data on WT Gag assembly platform diameters described in adherent COS cells^1,30^ and suggest that the assembly platform size is independent of the host cell type. However, except for Δp6, WT, WM and even NL4.3ΔPolΔEnv Gag presented very similar cluster sizes indicating that once platform assemblies are formed at the cell membranes, their size do not dependent upon CA-CA interaction, or on the presence of a packageable viral genome. The PALM images were then used to quantify the cluster density (i.e., the number of assembly platforms per cell surface units, Fig. 2b). It decreased from 4.5 ± 0.8 for WT Gag and 3 ± 0.6 clusters per *μm*^2^ for NL4.3ΔPolΔEnv Gag (mean ± C.I.) to 1.7 ± 0.9 clusters per *μm*^2^ for WM and to 0.05 ± 0.01 for MACASP1 Gag mutants.

This result suggests a strong role of the Gag C-terminus domain (NC-sp2-p6) in assembly platform formation in CD4^+^ T cells. Thus, PALM images of a Gag-Δp6 mutant (Fig. 2b, Δp6), which displays only the deletion of the p6 domain at the C terminus of Gag, were acquired. Δp6 mutant is a VLP release deficient mutant, forming groups of attached VLPs, as seen by electron microscopy (Fig. S2a) and by PALM imaging (Inset of Fig. 2b, Δp6). These grouped VLP lead to an overestimation of the Δp6 cluster mean diameters and an underestimation of their density (Fig. 2). Nevertheless, cells expressing Δp6 exhibited assembly platforms densities at least 20 times higher than the one observed for MACASP1 (Fig. 2d), revealing that the NC domain of Gag was the major determinant for generating high density membrane Gag clusters. On the opposite, the lack of p6 domain in Gag protein does not affect the cluster density.

### Monitoring how single Gag molecules are recruited at the budding site in living CD4^+^ T cells

**Figure 3:**
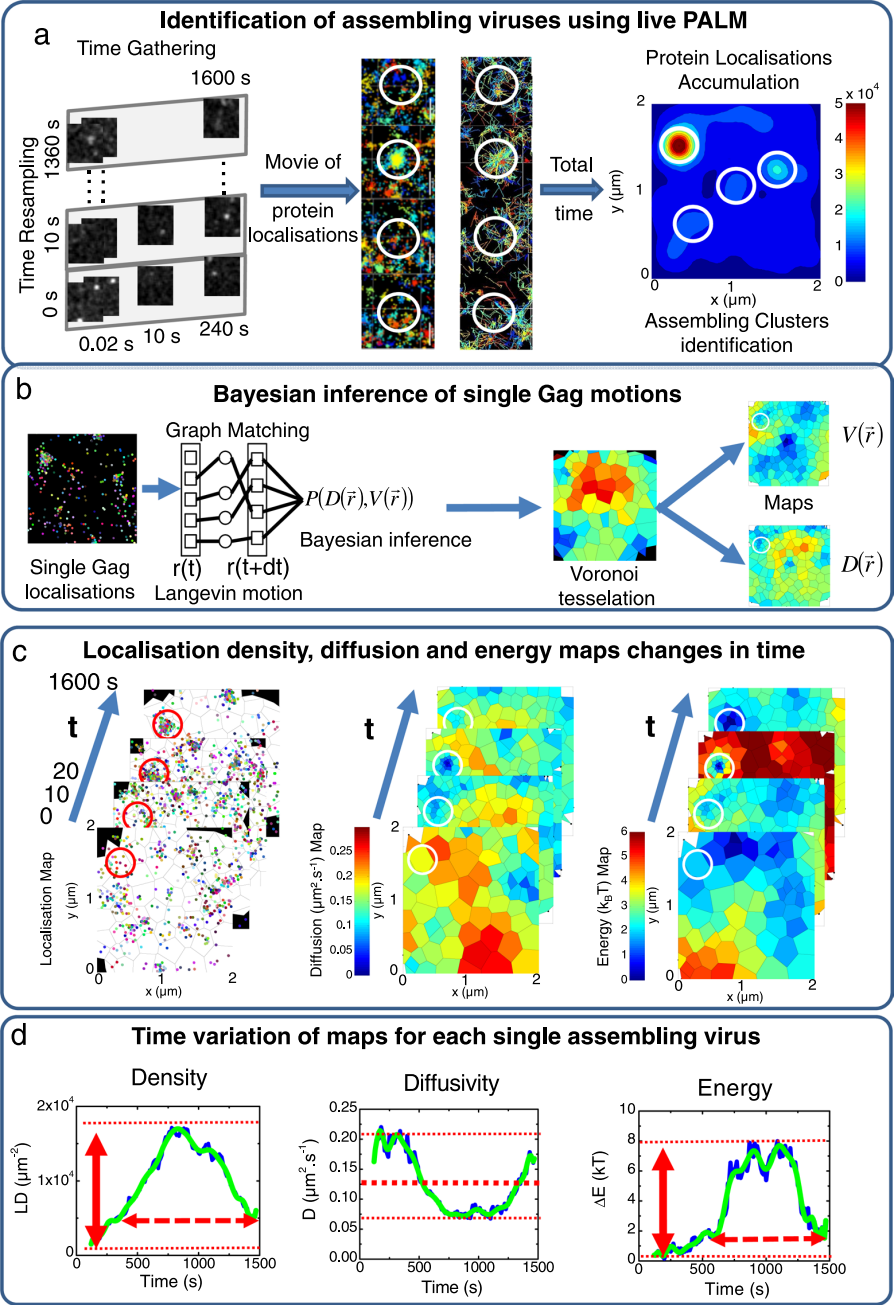
Monitoring time evolution of localisation densities, diffusivities and effective energies to characterise HIV-1 Gag(i)mEOS2 assembling VLP properties. (**a**) Single molecule localisations of each acquired image every 0.02 s are accumulated post experimentally for 240 s and gathered in a new image. This process is reproduced with a 10 s time shift until the end of the experimental acquisition (1600 s). Each newly generated image is used to make a movie illustrating changes of localisation over time. Here are shown typical examples of localisation changes over time during assembly of WT Gag and, as an illustration, typical trajectories observed around an assembling VLP. All trajectories seem to be directed towards the centre of the assembling VLP. Finally, projection of the images of this movie gives the total density accumulation in each experimental set of data, allowing identification of potential assembling clusters (white circles) for the analysis. (**b**) Localisations in each frame are reconnected using graph matching, an overdamped Langevin equation to describe molecular motion and Bayesian Inference. Using Voronoi tessellation, single-molecule images are divided in sub-areas containing the same number of localisations (n = 40) allowing for the generation of maps (see methods for details). (**c**) Maps of localisation densities, diffusion coefficients and effective energy are then generated for different times as in a), x- and y-axis values corresponding to the position in *μ*m inside the PALM image. (**d**) From these maps we extract plots of localisation density (LD, *μ*m^−2^, left panel), diffusion (centre panel) and effective energy (right panel) variations over time, during assembly of one VLP. From these these plots, the localisation density increase (LDI, red arrow, left panel) and the assembly time length (red dotted arrow, left panel) were quantified for each assembling VLP for WT Gag and mutants. The mean diffusion constant was also obtained (bold dotted line, centre panel) as well as the maximum trapping energy (red arrow, right panel) and the time length of trapping energy increase (dotted red arrow, right panel).

Live PALM was then used to monitor the changes in motion and densities of single Gag molecules in the vicinity of the assembling platforms. For that purpose, we created a movie with all the frames acquired in 1600 s (26 min). Each image of this movie included all the molecules localised in a 240 s (4 min) window with a sliding time of 10 s (Fig. 3a, left, middle and Fig. S4). We then accumulated images of the movie over time into one single frame in order to identify assembling events (Fig. 3a right). Figure 3a centre show the associated trajectories of single WT Gag molecules around a forming VLP (see also Video S1) obtained with a single particle tracking reconstruction algorithm. It can be seen that, in addition to diffusive motions, Gag trajectories directed towards the VLP centre were also observed, suggesting that VLPs in formation act as an energy trap for neighbouring Gag proteins.

Gag dynamics were analysed with a newly developed method that uses Bayesian inference to estimate physical parameters of motion^24^ in sub-domains (established by Voronoi Tesselation), in the vicinity (inside the circle in Fig. 3b), and far from budding sites. The overdamped Langevin equation was used to quantify the proportion of directed motion versus free diffusion in Gag motions (for details, see Methods, eq. 2). This directed motion was quantified by an effective trapping energy (E) that measures the strength of the Gag attraction towards the assembly site. We assert that the Gag protein is attracted when E is higher than 1 *kT*. Using this approach, localisation densities (LD), diffusion (D) and trapping energy (E) maps were built from the motions of all single Gag proteins detected during the 26 min movie (Fig. 3c). To monitor precisely VLP formation, the start time and the position of forming VLPs (assembling platforms, hereafter) were identified as the time and position where the LD was three times higher than in the surrounding area. Moreover, the apparent VLP radius was also measured and was defined as the distance were the LD was four times lower than at the VLP centre (see Methods for details). By multiplexing this approach, close to 2500 assembling platforms were analysed in 16 different cells for WT and mutant Gag proteins. Then, LD, D and E changes during the overall acquisition time were investigated (see Video S2 for examples of temporal changes of D and E maps) and were plotted (Fig. 3d) for each forming VLP. From these plots, the maximal LD increase (LDI, red arrow) and the overall particle formation duration (dotted red arrow) were measured (Fig. 3d left). It was previously shown^19^ that HIV-1 Gag assembly in adherent HeLa cells can be divided in three phases characterised, respectively, by an increase in LD (assembly), followed by a plateau value and then a decrease in LD (due to particle release). These three phases were also observed here in the host CD4^+^ T cells (Fig. 3d) and the assembly duration was measured (see Table S2).

The mean diffusion (bold dotted line in Fig. 3d centre, Methods, eq. 7) and the maximum trapping energy (ΔE, full red arrow in Fig. 3d right, Methods, eq. 8) were also extracted, as well as the duration of the trapping energy increase (dotted red arrow in Fig. 3d right).

**Figure 4:**
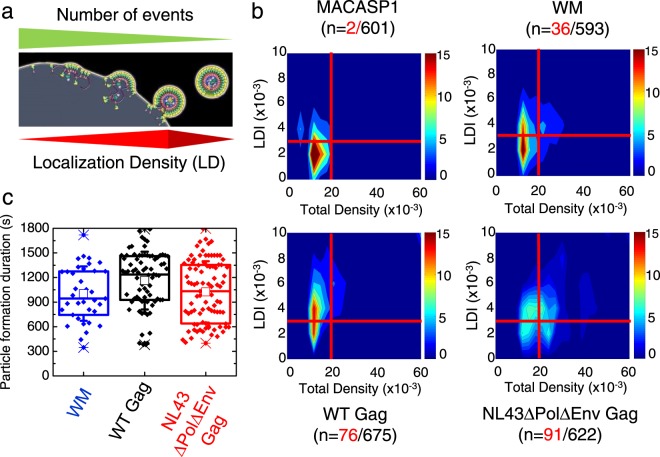
Maximal effective trapping energy and diffusion of Gag(i)mEOS2 (WT and mutants) in VLPs during their assembly at the plasma membrane of Jurkat T cells. (**a**) Schematic representation of VLP assembly showing the different steps and their relation with localisation density (LD) changes. The final step (before VLP release) is expected to be the step were localisation density increase (LDI) and the total density are the highest. It is also expected to be the less frequent one. (**b**) Diagram of LDI as a function of total density (localisation density accumulation during the time of acquisition) for WT Gag, the WM and MACASP1 mutants, and NL4.3ΔPolΔEnv Gag. Red lines delimit the threshold value used for both LDI and total LD to sort the fully assembling VLPs from the others; n: in black, number of VLPs before selection, in red, fully assembling VLPs (**c**) Box plot of VLP assembly and release duration of the previously selected fully assembling VLP made of WM (blue), WT Gag (black) or NL4.3ΔPolΔEnv Gag (red). The average duration for particle budding was 16 ± 2 min for WM (n = 36), 20 ± 2 min for WT Gag (n = 76) and 17 ± 1.5 min for NL4.3ΔPolΔEnv Gag (n = 91).

In a first attempt, we characterised the differences in behaviour of WT Gag and mutants independently of the assembling clusters by generating normalised distributions of the ΔE as a function of D values for the 600 assembly platforms identified in section 3 (Fig. S5). These distributions were compared to those obtained for CAAX(i)mEos2 as a negative control of self-assembly. These diagrams showed that the normalised distribution peaks were progressively drifting from the right for CAAX(i)mEos2 (non-assembling molecules) to the left for NL4.3ΔPolΔEnv Gag (assembling Gag molecules), i.e., from high to low diffusivity. Interestingly, WM molecules formed two populations characterised by a distribution similar to that of MACASP1 and WT molecules, respectively. Importantly, while the Gag mutants had spread distributions, NL4.3ΔPolΔEnv Gag always exhibited a quite narrow and fairly centred peak. In parallel, the diffusion(D)/localisation density increase(LDI) diagram show that increasing LDI lead to decreasing D, supporting the usefulness of these 3 parameters to quantitatively describe the assembly process. Surprisingly, the mean ΔE values (1–2 k_*b*_T independently of the condition) (Fig. S5) were not in favour of a strong attractive process during assembly. However, they were the mean of all “on-going” assembling VLPs, including those that will assemble imperfectly, i.e. not reaching the fully assembled and budding VLP state. Indeed, the total density (i.e., localisation density accumulation during the acquisition time) will reach its maximal value when VLPs are fully assembled, whereas the maximal value of localisation density increase(LDI) can occur in different situations (see Fig. 4a for a schematic representation). For example, an assembly platform not leading to VLP formation can have a high LDI value, but a low total density (e.g., MACASP1 in Fig. 4b). Conversely, a VLP almost fully assembled, suddenly appearing in the field of view, will have a low LDI, but a high total density.

To discriminate amongst these different possibilities, the localisation density increase (LDI) values were distributed as a function of the total density (localisation density accumulation) of every identified assembling platform (*n* > 600) for each mutant (Fig. 4b). Finally, to monitor correctly the formation of single VLPs in T cells, only isolated assembling platforms (i.e., separated by 400 nm, which is about six times the radius of a released VLP) were considered. We therefore explored threshold values for each parameter (LDI and total density value) in order to be in line with the relative assembling platforms densities observed in fixed cells (Fig. 2d) and the VLP production (Fig. 1c). Particles with high LDI (>2,500 *μm*^−2^) and total density values >20,000 *μm*^−2^ were selected (red lines on Fig. 4b) since only two of the 600 MACASP1 clusters identified were above these threshold values. Conversely, these thresholds allowed selecting 91 assembly platforms for NL4.3ΔPolΔEnv Gag, 76 for WT Gag and 36 for WM. The reduction by half of the number of assembly platforms for WM compared with WT Gag is in agreement with their VLP release data (Fig. 1c) and cellular densities (2d). First, we analysed the distribution of the VLP formation time values (Fig. 4c). The results indicated that the mean formation time (assembly and budding) was not significantly different for WT Gag, NL4.3ΔPolΔEnv Gag and WM (1160 ± 110 s, 1020 ± 80 s and 1003 ± 80 s, respectively). However, the individual values were very variable (from 350 s to 1800 s). Calculation of the duration of the first two phases (i.e., localisation density increase and plateau) showed that the first phase (assembly) lasted about 5 min for NL43ΔEnvΔPol Gag, 7 min for WT Gag and 6 min for WM. The plateau phase duration was about 6 min for all three Gag derivatives (the exact values are in Table S2). Altogether these results suggest that: i) MACASP1 forms low-density assembling platforms that mainly do not reach the VLP formation stage, ii) only WM and WT Gag can form high-density assembly platforms that lead to VLP formation, iii) on average, the time needed to make a VLP seems not affected by the presence of a packageable viral RNA or by CA-CA interactions.

### The viral genome is spatio-temporally coordinating the recruitment of Gag molecules at the budding site

**Figure 5:**
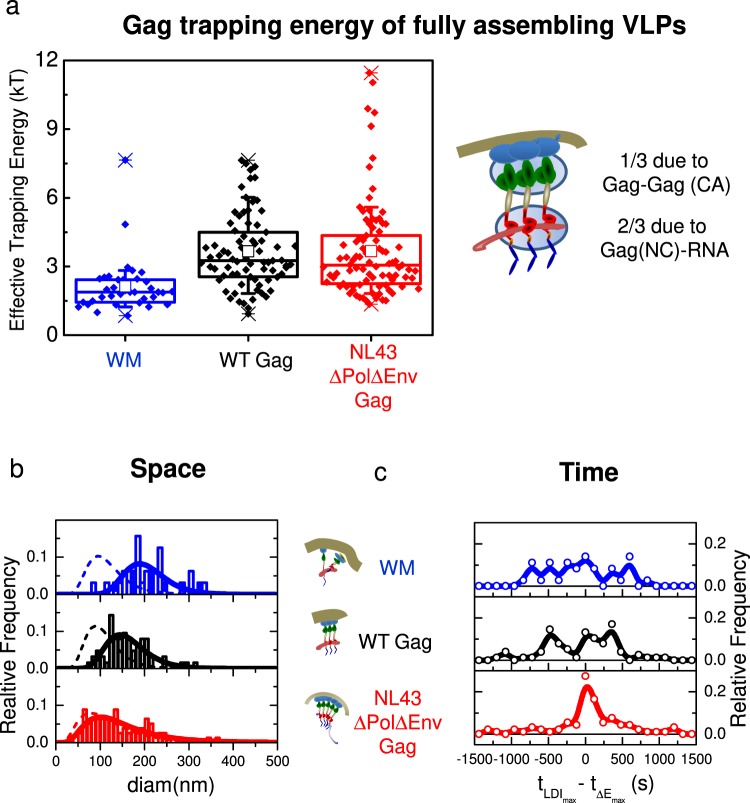
The spatio-temporal coordination of membrane Gag assembling VLPs. (**a**) Box plot of the maximum effective trapping energy observed in fully assembling VLPs. (**b**) Normalised distributions (bars) and their log-normal fits (plain lines) of the diameters at full width at half maximum (FWHM) of the maximal intensity attractive potentials for WM (blue), WT Gag (black) and NL4.3ΔPolΔEnv Gag (red). Dashed line represents the log-normal fit of the assembling clusters distribution observed in fixed cells and depicted in Fig. 2. (**c**) Normalised distributions of the difference between the time to reach the maximum localisation density increase (LDI) and the time to reach the maximal energy trapping for WM (blue), WT Gag (black) and NL4.3ΔPolΔEnv Gag (red). Distributions were significantly different according to the Kolmogorov-Smirnov test (see Table S4 for values).

After selecting only the population of fully assembling VLPs (Fig. 4b), we quantified the effective trapping energy for each Gag and derivatives condition (Fig. 5a). The only two selected MACASP1 particles exhibited no difference in effective trapping energy values compared with the total pool shown in Fig. S5 (ΔE = 1.5 ± 0.1 *k*_*b*_*T*), whereas, the maximal effective trapping energy value slightly increased for the subset of VLP generated by WM (ΔE = 2.1 ± 0.4 *k*_*b*_*T*). Conversely, the effective trapping energy value was strongly increased for WT Gag (ΔE = 3.7 ± 0.4 *k*_*b*_*T*) in comparison with WM (p < 5.10^−5^, Student’s t test, see Table S3 for exact values of p). Overall, these data indicate that a lack of correct CA-CA interactions during assembly induced a decrease of 35% in effective Gag trapping energy and suggest that the 65% left of the trapping energy are mainly due to the RNA binding-NC domain of the Gag protein. Interestingly, the presence of either the packageable viral RNA, or a Rev/RRE Gag dependent sequence (NL4.3ΔPolΔEnv), did not influence the mean effective Gag trapping energy value (ΔE = 3.7 ± 0.4 *k*_*b*_*T*) questioning the role of the viral RNA in the assembly process.

It has been previously shown that molecular self-assemblies are more efficient and are spatially controlled when triggered by heterogeneous seeds, i.e., by protein-RNA interactions rather than protein-protein interactions^31–33^. We hypothesised that Gag(NC)-viral RNA interaction acted here as the most efficient seed to spatially and temporally coordinating Gag self-assembly. To test this hypothesis, we first compared the attraction distance in the late steps of the assembly process, for the different derivatives of Gag (Fig. 5a). The mean values of these attraction distance decreased from 207 ± 20 nm for WM to 162 ± 13 nm for WT Gag and finally to 140 ± 17 nm for NL4.3ΔPolΔEnv Gag. Moreover, when these attraction distance distributions (plain line in Fig. 5a) were compared to the distribution of assembling clusters diameter (dotted line in Fig. 4e) obtained by PALM (Fig. 2c), we observed the strongest overlap for NL4.3ΔPolΔEnv Gag. This indicates that when the packageable *Psi*-containing viral RNA genome is present the trapping potential is spatially controlled and narrowed, ending to an efficient proportion of fully assembled VLPs over assembly clusters. Finally, in order to test the temporal coordination of the VLP assembly process, the time to reach the maximum localisation density increase (LDI) was compared to the time to reach the maximal effective Gag trapping energy intensity, for WM, WT Gag and NL4.3ΔPolΔEnv Gag. Figure 5b shows the significantly different distributions of these differences in time (detailed statistics in Table S4). Interestingly, they were dispersed both for WM and WT Gag, whereas NL4.3ΔPolΔEnv Gag showed one major peak centred on 0 (Fig. 5c) indicating a perfect temporal coordination of Gag assembly in this case.

These important results show that in host *CD*4^+^ T-cells, the presence of the packageable viral RNA genome, which also contains a wild-type Gag Rev-dependent coding sequence, first acts as a seed and then control the temporal and the spatial coordination of viral Gag protein self-assembly.

## Discussion

In the last ten years, many efforts have been made to study the assembly of HIV-1 Gag particles in living cells^18–21,34^. Here, we monitored immature HIV-1 Gag assembly dynamics at the plasma membrane of host CD4^+^ T lymphocytes using quantitative live PALM imaging coupled to advanced big data quantitative analyses. Using different known Gag assembly defective mutants we highlighted the respective role of CA-CA, NC-RNA and p6-ESCRT interactions in membrane attached Gag clusterisation and VLP formation. First, using PALM imaging on fixed cells, our results showed that assembly platform densities were quite different among the Gag derivatives, reflecting which domain of Gag was necessary for membrane binding and assembly (Figs 2 and S3). MACASP1 molecules showed the most drastic assembly platform density reduction (by 50-fold compared with WT Gag) while WM exhibited only a 3-fold reduction. This suggested that the assembly efficiency at the T cell membrane strongly depends on Gag C-terminal domains (NC-sp2-p6), but not on the CA-SP1 interface alone. This is in good agreement with the work of Robinson *et al*.^35^, showing that a MACA mutant is unable to produce high order multimers of Gag using velocity sedimentation and gradient assays. It also showed that the helical bundle of the CA-SP1 junction segments, as a distinctive interaction stabilising the immature lattice^36–38^, was only effective when the NC domain was present, suggesting that the NC domain of Gag, certainly via its interaction with RNA, was mandatory for initiating membrane Gag stabilisation and assembly. Furthermore, the 20-fold higher density for Δp6 compared with MACASP1 suggests that a direct effect of ESCRT protein (such as Tsg101-p6 interaction) on assembly platform formation is unlikely. VLP formation analysis (compare Video S3 and S2 for Δp6 and WT Gag, respectively) showed that Δp6 Gag molecules can assemble at the T cell plasma membrane, but accumulate and remain in the same location. Gag Δp6 VLP persistence at the plasma membrane most probably indicates a defect in particle release as shown by electron microscopy (ref.^15^ and Fig. S2 here for Δp6 Gag(i)mEOS2). We thus propose that the remaining NC domain is the main determinant for Gag assembly platform formation at the cell membrane of CD4^+^ T cells. Most probably the Gag molecules trapping occur via NC-RNA interactions that induce and stabilise Gag-Gag multimers at the cell plasma membrane.

To go further, we performed live PALM and quantified single-molecule dynamics by analysing the trajectories of individual membrane Gag molecules (the cytosolic ones could not be monitored by this approach) in order to (1) measure the kinetics of immature HIV-1 VLP formation at the surface of host T cells, and (2) determine how individual Gag molecules sensed the viral bud in formation.

First, we focused on the single Gag molecule localisation density changes over time to localise viral particle in formation and to decipher their kinetics. We observed three different phases (increase, plateau and decrease) in the formation and release of newly assembled particles, as previously described in adherent HeLa cancer cell lines by classical TIRF microscopy. We observed for WT Gag and NL4.3ΔPolΔEnv Gag, that the first phase (considered the assembly phase) lasts approximately 5 min, in agreement with the value found by Jouvenet *et al*.^18^ but lower than what was reported by Ivanchenko *et al*.^19^ (both in adherent HeLa cells). The second phase (plateau phase) lasted approximately 10 min, as observed in HeLa cells^19^, and preceded a decreasing third phase. It is noted that in the case of Δp6, the process stops at the plateau (Video S3), confirming that this plateau correspond to the ESCRT machinery recruitment^39^. We did not quantify the third phase because it was quite variable, possibly due to many different processes (pinching off, bleaching). Instead, we measured the total time of particle formation as the time length between the appearance and disappearance (i.e., return to the initial localisation density) of an assembly platform (see Video S1). The mean duration was observed between 17 min (for NL4.3ΔPolΔEnv) and 20 min (for WT Gag), but with high variability (7 to 30 min), as already observed in HeLa cells^39^. This showed that the presence of the packageable viral RNA genome did not significantly decrease the total time for VLP assembly and release. Our and previous results^19,39^, show that assembly duration and the time needed to achieve a fully released particle seem to be independent of the cell type.

Then, we furthermore questioned how each individual Gag were recruited to the assembly site. While visualising individual Gag molecule trajectories, we observed that Gag tended to move towards the centre of assembling particles, suggesting the existence of an attractive potential at the origin of Gag recruitment into the assembling site. The live PALM data were then analysed using Bayesian inference and the modified Langevin equation to quantify the motion of individual Gag molecules^24,25,40^. Using the Langevin description of the motion, the key dynamical properties were approximated as diffusion and effective trapping energy maps, providing us with a more general understanding of the modifications of protein dynamics at the vicinity of assembling platforms. Therefore, by computing temporal maps of Gag diffusion and attractive properties in and around the assembling platforms, we measured the intensity and the spatial range of this attracting energy. We obtained the highest intensity for NL4.3ΔPolΔEnv Gag and WT Gag ($< {\rm{\Delta }}E >  \sim 4{k}_{b}T$). Recently, using coarse grained molecular simulation Pak *et al*.^41^ has estimated that the oligomerisation via CA-CA hexamer formation due to SP1 interactions in the presence of (simulated) membrane and RNA could occur for weak SP1-SP1 interaction energy (∼4 *k*_*b*_*T*). Interestingly, this value is exactly in line to what we found for WT-Gag in Jurkat T-cells. This value was reduced by 35% for the CA-CA(WM) mutant molecules that still generate platforms and was down to 1.5 *k*_*b*_*T* (65% reduction) for MACASP1 molecules that do not generate VLPs. This last value is close to the thermal fluctuation energy (1 *k*_*b*_*T*) and therefore, typical of an energy that induced no Gag trapping. This show that two-third of the Gag trapping is due to RNA-NC domain interaction while the rest corresponds to direct Gag-Gag interaction.

Nevertheless, the mean effective Gag trapping energy we measured cannot only reflect direct protein-protein or protein-RNA interaction as suggested by the long spatial range (50 *nm* < *r* < 100 *nm*, i.e., 1 to 2 average bud diameter) observed here for the attractive forces. Indeed, the CA-CA interaction defective mutant (WM) still generates VLPs in CD4^+^ T-cells although exhibiting a <Δ*E*> value lower than 4 *k*_*b*_*T*. Several reasons can account for this long distance recruitment observed here. The most obvious is the presence, at the budding site, of an RNA (viral or not) that can bind Gag proteins far from the VLP centre and act as the long distance attractor. But the cell plasma membrane can also play a role in this long range potential. Indeed, long range attractive forces such as thermal Casimir forces has recently been shown to be responsible for Shiga Toxin clustering on membranes^42^. Previously, Sens *et al*.^43,44^ theoretically predicted for caveolae that, when the force exerted on the plasma membrane by oligomers decreases, the resulting bud radius is proportionally increased. In our study, the almost two-fold reduced Gag attractive energy when CA-CA interaction were loose (WM) and its two fold enlarged attraction size was correlated with the existence of an increase in the WM-VLP diameter compared to the WT as observed by electronic microscopy (Fig. S2). This suggests that in Jurkat T-cells, the plasma membrane can rescue the lack in efficient CA-CA dimerisation in order to produce immature particles, as already observed on model membranes^10^. This is also in good agreement with the labile membrane bound form of WM Gag mutant observed in 293T HEK cells in the Robinson *et al*.^35^ biochemical and EM study and in our membrane flotation assay (Fig. S3).

Finally, neither the presence of the assembly-triggering Psi RNA sequence, nor the Rev/RRE driven RNA trafficking or Gag sequence changed the Gag attraction intensity towards the assembly site. Therefore, to gain more insight into the role of CMV-driven codon optimised Gag (WT Gag and WM) versus the cis packageable Rev/RRE driven viral Psi containing RNA genome (NL4.3ΔPolΔEnv Gag), we analysed the temporal correlation between the Gag density and the Gag attraction energy increase. Unlike WT Gag and WM, a perfect temporal correlation between Gag density and Gag trapping energy was observed for NL4.3ΔPolΔEnv Gag. This result reveals an important role for the Psi/RRE containing viral RNA genome in the spatio-temporal coordination of HIV-1 Gag assembly at the plasma membrane of CD4^+^ T cells. This can be either due to the specific interaction of the genomic RNA with the NC domain of Gag during Gag multimerisation at the cell membrane, or to the fact that the coordination of virion assembly depends on the Rev/RRE genomic RNA intracellular trafficking pathway. The first hypothesis is in good agreement with the fact that the viral RNA genome (containing the Psi signal for encapsidation) acts as a structural element for retroviral particles^45,46^ and with the model recently proposed by Chen *et al*.^27^ showing that miRNA binding to the NC domain of Gag inhibits HIV-1 assembly. Furthermore it was described that high-order Gag multimerisation only occurs at the cell membrane^47^ and is dependent on Gag membrane-binding capacity. Here, our results suggest that the formation of high-order membrane Gag multimers depends strongly on NC domain of Gag. The second hypothesis goes with the recent work of Becker and Sherer showing that the viral mRNA subcellular trafficking and location regulates viral assembly at the cell membrane^21^.

## Conclusion

**Figure 6:**
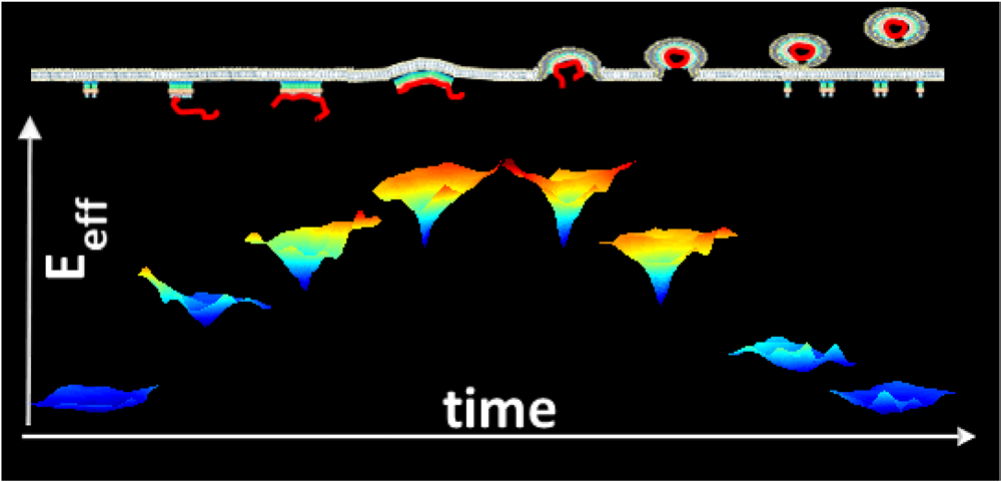
A schematic view of change in effective Gag trapping energy during HIV Gag assembly and budding. The top scheme represents Gag molecules oligomerisation at the cell plasma membrane (in grey) with the viral RNA genome (in red). Below is represented the color coded (increasing from blue to red) effective attractive energy well. Deepest depth represent the highest energy difference.

In conclusion, using quantitative single-molecule localisation microscopy in living CD4^+^ T cells, we measured how each single membrane Gag was recruited to HIV-1 assembly sites. Our results can be summarised in the schematic illustration (Fig. 6) showing the time evolution of the Gag recruitment energy at the assembly site during the bud formation. We also showed that the presence of a packageable cis-acting viral RNA genome, coding for Gag, induced a spatio-temporal coordination of immature HIV-1 assembly at the plasma membrane of host CD4^+^ T cell. This strongly suggests that the fate of the viral RNA genome (trafficking or encapsidation) and its interaction with the nucleocapsid domain of Gag synchronises Gag assembly at the plasma membrane of host CD4^+^ T cells.

## Methods

### Cell culture

Jurkat T cells (a human T-cell leukaemia cell line, ATCC-CRL-2899TM) were grown in RPMI 1640 plus Glutamax (Gibco) supplemented with 10% foetal calf serum (FCS) and antibiotics (penicillin-streptomycin. Human embryonic kidney (HEK) 293T cells (ATCC-CRL-1573TM) were grown in DMEM (Gibco) supplemented with 10% FCS and antibiotics (penicillin-streptomycin).

### DNA plasmids

The plasmid expressing HIV-1 Gag with the internal (located between MA and CA) mEOS2 tag fused with the Gag protein, called pGag(i)mEOS2 WT (CMV promoter-driven codon optimised Gag sequence), was used to generate the WM and MACASP1 mutants by site-directed mutagenesis. Another plasmid that expresses HIV-1 WT Gag(i)mEOS2 and the viral cis-packageable Psi RNA genome with a Rev/RRE dependent Gag coding sequence (pNL4.3ΔPolΔEnv) was kindly provided by Dr Eric Freed (NIH, Frederick, MD, USA), and was described previously^27^.

### Site-directed mutagenesis

Mutations were introduced in pGag(i)mEOS2 WT by site-directed mutagenesis using the QuickChange mutagenesis kit (Agilent) according to the manufacturer’s protocol. Tryptophan 184 and methionine 185 were replaced by two alanine residues (WM mutant, as in^48^) using the primer 5′-GAC GTG AAG AAC GCA GCT ACC GAG ACC CTG-3′. NC-sp2-p6 were deleted by inserting a stop codon after the sp1 sequence (MACASP1 mutant) using the primer 5′-GCG ACC ATC ATG TAG CAG CGC GGC AAC-3′. The p6 sequence was deleted by inserting a stop codon after the sp2 sequence (Δp6 mutant) using the primer 5′- CCC GGC AAC TTC TAG CAG AGC CGC CCC-3′. All plasmids were amplified in E. coli and mutations were confirmed by DNA sequencing (MWG Eurofins).

### DNA transfection

Jurkat T cells (2 × 106) were microporated with 4 *μ*g of each plasmid using the Amaxa system (Lonza) and then plated in RMPI complete medium and harvested 24 h post-transfection, as described in^28^. HEK293T cells were transfected by using the calcium phosphate buffer, as described in^49^.

### Cell viability, transfection efficiency and protein expression by FACS

Cell viability, cell transfection and protein expression were assessed with a BD FACS Calibur flow cytometer. FACS results were analysed with the FlowJo software v10. The cell viability rate was calculated as the ratio of cell size and granulometry over the total cell number. Protein expression rates were monitored using the geometric mean of the mEOS2(+) cell fluorescence intensity distribution. Antibodies. Western blots were performed using the anti-CAp24 (NIH AIDS Reagent Program HIV-1 p24Gag monoclonal (24-4) mouse antisera) and mouse anti-LAMP2 (human lysosome-associated membrane protein 2) (H4B4) (Santa Cruz Biotechnologies) antibodies, followed by anti-mouse and anti-rabbit antibodies coupled to horseradish peroxidase (HRP) (Dako), and an anti-GAPDH HRP-coupled antibody (Abcam).

### VLP purification and immunoblotting

To monitor viral particle production, cell culture media containing VLPs were harvested at 24 h post-transfection. Jurkat T cell supernatant was clarified by centrifugation at 60000 g for 10 min, while HEK293T cell supernatants were filtered (0.45 *μ*m pore size). Viral supernatants were purified by ultracentrifugation through a sucrose cushion [25% glucose (wt/vol) in TNE buffer (10 mM Tris-HCl pH 7.4, 100 mM NaCl, 1 mM EDTA)], at 100000 g in a Beckman MLA150 or SW60Ti rotor for 90 min. Ellets were resuspended at 4 °C in TNE buffer overnight and stored at −80 °C. To analyse the intracellular viral protein content, cells were lysed in RIPA buffer (150 mM NaCl, 20 mM Tris-HCl [pH 8], 1% NP-40, 0.1% SDS, 0.2 mM EDTA) and sonicated. Cell lysates were then clarified at 16000 g for 10 min, and the protein concentration in the cell lysates was determined with the Bradford assay. For western blot analysis, 50 *μ*g of total proteins were loaded and separated on 10% SDS-PAGE gels and transferred onto polyvinylidene difluoride membranes (Thermo Fischer). Immunoblotting was performed using the relevant antibodies and HRP signals were revealed with the SuperSignal West Pico substrate (Thermo Scientific). Transfection efficiency and VLP release calculation. Plasmid transfection efficiency in T cells was evaluated by measuring the percentage of fluorescent cells by immunofluorescence or flow cytometry analysis. For VLP release, the HRP signals from immunoblot membranes were imaged using the G:Box system (Syngene), and the viral Gag or CA protein signals were quantified using the ImageJ software. The percentage of VLP release relative to the GAPDH loading sample was then estimated as:

1 $$ \% VLP=100.\frac{({I}_{VS}-{I}_{blank})}{[(\frac{{I}_{CE}}{{I}_{GAPDH}}-{I}_{blank})+({I}_{VS}-{I}_{blank})]}$$

where *I*_*VS*_ is the viral supernatant (quantification of the immunoblot signals for Gagp55 and CAp24); *I*_*CE*_ is the quantification of the Gagp55 signal in cell extracts; and *I*_*blank*_ is the background signal. GAPDH served as loading control.

### Statistics

When given in the text or shown in the figures, data are represented as mean values plus or minus confidence interval (C.I.). C.I. represents 1.96 times the standard error of the mean (S.E., $S.E=\frac{S\mathrm{.}D\mathrm{.}}{\sqrt{n}}$, with n = sample size), corresponding to the upper and lower 95% confidence limits (i.e., *α* = 0.05). Depending on the nature of the data, different statistical test were performed. When the mean values were to be compared, Student’s t test were performed directly on the values (for normal distributions) or on their log (for log-normal distributions). The results of the Student’s t-test obtained here were furthermore confirmed with Wilcoxon rank sum test (unknown distribution). Finally when two distributions were compared, the non parametric Kolmogorov-Smirnov test was used. All the detailed values of the p-values obtained for all the test performed (except Wilcoxon rank sum test) are given in Tables S1, S3, S4 of the SI. The test were performed with MATLAB.

### Photo Activation Localisation Microscopy

#### Sample Preparation

To increase cell adhesion, Jurkat T cells were seeded on polylysine-coated coverslips and left in culture medium at 37 °C, 5% CO2 for 30 min. After rinsing with PBS, cells were incubated at 37 °C in microscopy buffer (MB) (150 mM NaCl, 20 mM HEPES pH7.4, 1 mM CaCl2, 5 mM KCl, 1 mM MgCl2 pH 7.4 and 100 nm TetraSpeck^TM^ microspheres) for live PALM experiments, or fixed with 3% PFA in PBS at room temperature for 15 min for PALM experiments. In this case, after fixation, cells were rinsed with 50 mM NH4Cl for 5 min and then several times with PBS before transfer in MB for microscopy analysis.

#### Live PALM and PALM image acquisition

Cells were imaged at 37 °C in Ludin chambers (Life Imaging Services) with an inverted motorised microscope (Nikon Ti) equipped with a 100 × 1.45 NA PL-APO objective and a perfect focus system for long acquisition under TIRF illumination. For live PALM, cells that express mEOS2-tagged Gag molecules were photoactivated using a 405 nm laser (Omicron) and the resulting photoconverted single-molecule fluorescence was excited with a 561 nm laser (Cobolt Jive^TM^). Both lasers illuminated the sample simultaneously. The photoactivation laser power was adjusted to keep the number of the stochastically activated molecules constant and sparsely distributed during the acquisition to allow single-molecule localisation (46). Fluorescence signals were collected by using a dichroic and an emission filter (F38-561 and F39-617, respectively, Semrock) and a sensitive EMCCD camera (Evolve, Photometric). Acquisition was guided by the MetaMorph software (Molecular Devices) at 50 Hz in streaming mode and analysed online with laser feedback to ensure the optimal and constant number of localisations during acquisition (46). Multicolour fluorescent 100 nm TetraSpeck^TM^ microspheres (Invitrogen) were used as fiduciary markers to acquire and correct for lateral drifts occurring during long-term acquisitions. LivePALM experiments allowed the acquisition of sets of 80,000 images per cell that were analysed with WaveTracer (Molecular Devices) and the custom-made PALM Tracer analysis software to extract molecule localisation and dynamics data. Fluorescent single molecules were localised and tracked over time using a combination of wavelet segmentation and simulated annealing algorithms^50,51^, operating as a plug-in for the MetaMorph software (Molecular Devices). Using the same experimental conditions described above, the system resolution was quantified to 46 nm at full width and half maximum using fixed mEOS2-expressing cells. 200 two-dimensional distributions of single-molecule positions belonging to long trajectories (>30 frames) were analysed by bi-dimensional Gaussian fitting. The resolution was defined as 2.3 *σ*_*xy*_, where *σ*_*xy*_ was the standard deviation of the Gaussian fit.

Assembling platform apparent diameter were determined as the 1/*e*^2^ diameter of a bi-dimensional Gaussian fitting.

#### Robust statistical Gag dynamics analysis using Bayesian inference

The large amount of data and the time-evolving nature of the process required an automated and stereotypical way to analyse data. Thus, all data were analysed exactly in the same manner, including the inference hyper-parameters. A pipeline was designed for all data analysis. The pipeline included five steps:

- Single Molecule Localisation
- Non-tracking with Graph Assignment
- Selection of the Regions of Interest
- Time-Evolving Bayesian Inference Analysis
- Time-Evolving Feature Extraction from Inferred Maps

Single-molecule localisation was implemented in MATLAB using the slightly modified MTT algorithm^52^. To limit errors due to tracking algorithms, we did not track single molecules, but used optimal assignments between consecutive images to extract Gag movements. Region of interest (ROI) selection was then based on LDs (localisation densities). ROIs were selected as squared areas of 2 *μm* in length centred on the maximum of density. The number of ROIs per cell was limited to 30. In each ROI, the effective centre of a VLP, *r*_*eff*_, was defined as the point with the highest LD (${\rho }_{{\max }}^{tot}$) (cumulated on the 80 000 frames). The effective radius, *R*_*eff*_, of a VLP was defined as the average distance between *r*_*eff*_ and the points of a density equal to (${\rho }_{max}^{tot}/4$). In the analysis, points within *R*_*eff*_ were considered to be in the VLP. Depending on the density of maturing VLPs, more than one VLP can be present in a single ROI. All VLPs inside such region were analysed. They were discarded afterwards by LD/LDI selection (Fig. 4). Then, the molecule motion was analysed using Bayesian inferences (see detailed Methods in supporting information for details). Briefly, the dynamics of individual Gag proteins were approximated with the Overdamped Langevin Equation (OLE) written using an Itô interpretation:

2 $$\frac{dr}{dt}=-{D}_{t}(r)(\frac{\nabla {V}_{t}^{eff}(r)}{{k}_{b}T}-\frac{\nabla {D}_{t}(r)}{{D}_{t}(r)})+\sqrt{2{D}_{t}(r)}\xi (t)$$

where *D*_*t*_(*r*) was the space-varying diffusivity, ${V}_{t}^{eff}(r)$ the effective potential, and *ξ*(*t*) the zero-averaged Gaussian noise. (${D}_{t}(r),{V}_{t}^{eff}(r)$) were considered to be the statistical features that encode the dynamical characteristics of the environment concerning the individual dynamics of Gag proteins. Bayesian Inference was used to extract (${D}_{t}(r),{V}_{t}^{eff}(r)$) from the assignment between images^24,53–56^ (see Supporting Information Methods for details of the Bayesian procedure). In eq. 2, the index t shows that diffusion and potential fields can change with time, but at a time scale larger than that of the particle dynamics. Nevertheless, the time-evolving dynamics during VLP assembly led to high variability in particle density. Thus, the tessellation procedure described in^24^ was modified to ensure more homogeneity in the structure of the Voronoi spatial tessellation (details in Supporting Methods). Finally, temporal changes in VLP dynamics were monitored by time windowing. The duration of the window was set to 240 s with a sliding time of 10 s. The inference was performed independently for each time window, allowing the map to be inferred independently of previous or future detections. Hence, the typical time-evolution of VLP maturation led to 136 maps of (${D}_{t}(r),{V}_{t}^{eff}(r)$). VLP density inside a map (corresponding to a time window) was evaluated as

3 $$\rho =\frac{N(t)}{\pi {R}_{eff}^{2}},$$

where *N*(*t*) was the number of localisations in the VLP during the time window t. The time-evolution of density for a VLP was directly computed as the density measured on the set of maps associated with that VLP. Considering that the set of mesh subdomains in the VLP at the time window t is *I*(*t*) and neighbours to a VLP are defined as the set of mesh subdomains in contact with the VLP and referred as *M*(*t*), then, at each time window t, the diffusivity in the VLP was defined as

4 $${D}_{in}(t)= < D{ > }_{I(t)},$$

and the VLP trapping energy was defined as

5 $$\delta {V}^{eff}(t)= < {V}^{eff}{ > }_{{M}_{2{R}_{eff}(j)}}-mi{n}_{I(j)}({V}^{eff}),$$

where <.> was the spatial average.

Finally, for each VLP present in the analysed ROIs (i.e., 600 VLPs per mutant), the time-evolving density, diffusivity and potential were smoothed with a 10th order Savitsky-Golay filter to extract the following parameters:

- Localisation Density Increase (LDI):
  6 $$LDI={\rho }_{max}-{\rho }_{min}$$
  - Mean diffusivity in the assembly platform:
    7 $$D= < {D}_{in}{ > }_{t}$$
    where <.> was the time average.
  - Maximum Trapping Energy:

8 $${\rm{\Delta }}E=\delta {V}_{max}^{eff}-\delta {V}_{min}^{eff}$$

The assembly time length was defined as the 1/*e*^2^ width of the Gaussian fit of the LDI peak. As the analysis led to ≃100,000 maps of (${D}_{t}(r),{V}_{t}^{eff}(r)$), the (${D}_{t}(r),{V}_{t}^{eff}(r)$) values observed in each VLP were distribute in classes. Each class was renormalised to the total VLP number. This allowed generating (*V*^*eff*^ = *f*(*D*), *D* = *f*(*LDI*)) diagrams for each mutant.

## Electronic supplementary material

Supporting Information

Single-particle trajectories of HIV-1 WT Gag(i)mEOS2 (left) and their accumulation (right) during WT Gag assembly at the Jurkat T-cell plasma membrane

Temporal changes of potential energy (right) and diffusivity (left) maps during HIV-1 WT Gag assembly, particle formation and release (This map is extracted from a selected area of the video S1)

Temporal changes of potential energy (right) and the diffusivity (left) maps of an already assembled, but unreleased, Δp6 mutant.
