# Author Correction: Single molecule localisation microscopy reveals how HIV-1 Gag proteins sense membrane virus assembly sites in living host CD4 T cells

**DOI:** 10.1038/s41598-018-35954-8

**Published:** 2018-11-22

**Authors:** Charlotte Floderer, Jean-Baptiste Masson, Elise Boilley, Sonia Georgeault, Peggy Merida, Mohamed El Beheiry, Maxime Dahan, Philippe Roingeard, Jean-Baptiste Sibarita, Cyril Favard, Delphine Muriaux

**Affiliations:** 1 Infectious Disease Research Institute of Montpellier (IRIM), UMR9004 CNRS, University of Montpellier, 1919 route de Mende, 34293 Montpellier, France; 2 Decision and Bayesian Computation, UMR 3571 CNRS, Pasteur Institute, Paris, France; 3 INSERM U966 and IBiSA EM Facility, University of Tours, Tours, France; 4 Light and Optical Control of Cellular Organization, Curie Institute, UMR, 168 CNRS Paris, France; 5 Interdisciplinary Institute for Neuroscience, UMR 5297 CNRS, University of Bordeaux, Bordeaux, France

Correction to: *Scientific Reports* 10.1038/s41598-018-34536-y, published online 02 November 2018

Three supplementary videos were omitted from the original version of this Article. This error has been corrected in the HTML version of the Article; the PDF version was correct at time of publication.

