# Supplementary material for: Single molecule localisation microscopy reveals how HIV-1 Gag proteins sense membrane virus assembly sites in living host CD4 T cells
Source: Sci Rep. 2018 Nov 2;8:16283. doi: 10.1038/s41598-018-34536-y (PMC6214999; doi:10.1038/s41598-018-34536-y)
Supplement: Supplementary file 1 — Supporting Information [file 41598_2018_34536_MOESM1_ESM.pdf]

Supporting Information for: Single molecule localisation microscopy reveals how HIV-1 Gag proteins sense membrane virus assembly sites in living host CD4 T cells.

Charlotte Floderer[1,+], Jean-Baptiste Masson[2,+], Elise Boilley[1], Sonia Georgeault[3], Peggy Merida[1], Mohamed El Beheiry[4], Maxime Dahan[4], Philippe Roingeard[3], Jean-Baptiste Sibarita[5], Cyril Favard[1,*], and Delphine Muriaux[1,*]

[1]Infectious Disease Research Institute of Montpellier (IRIM), UMR9004 CNRS, University of Montpellier, 1919 route de Mende 34293 Montpellier, France.
[2]Decision and Bayesian Computation, UMR 3571 CNRS, Pasteur Institute, Paris, France
[3]INSERM U966 and IBiSA EM Facility, University of Tours, France.
[4]Light and Optical Control of Cellular Organization, Curie Institute, UMR 168 CNRS, Paris, France
[5]Interdisciplinary Institute for Neuroscience, UMR 5297 CNRS, University of Bordeaux, Bordeaux, France.
[*]cyril.favard@irim.cnrs.fr, delphine.muriaux@irim.cnrs.fr
[+]these authors contributed equally to this work

Supporting Information

The supporting information contains 5 figures, 3 videos, 4 tables and detailed methods.

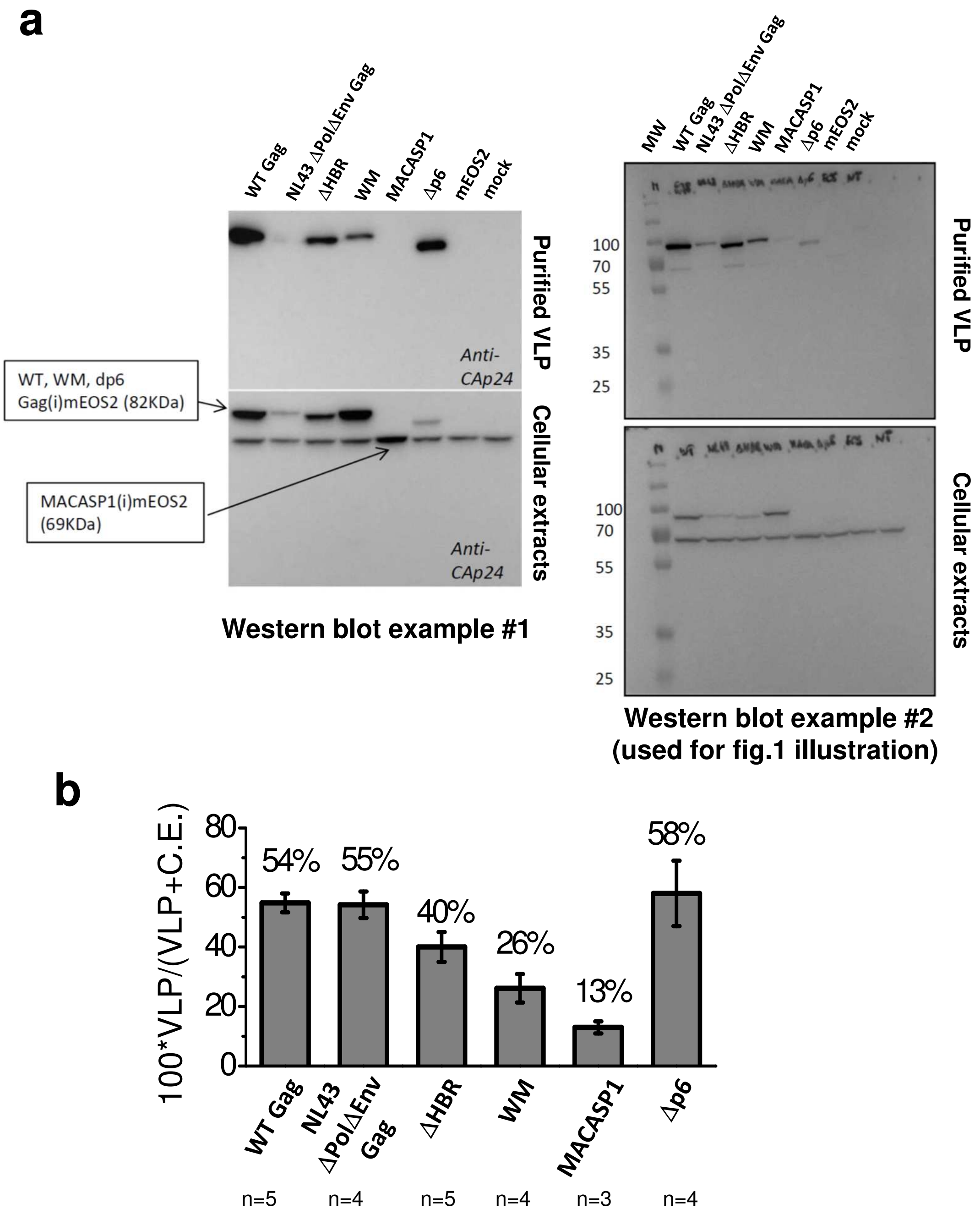

Fig. S1 Example of Immunoblots (entire gels) of Gag(i)mEOS2 wild type and mutants expressed in transfected Jurkat T cells and full quantifications (a) Two Examples of Western blots (entire gels) of Gag(i)mEOS2 wild type and mutants. Western blot analysis of HIV-1 WT Gag(i)mEOS2 protein and mutants expression in Jurkat T cells ("cellular extract", 50µg/well) and in purified VLPs ("VLP") revealed by mouse anti-CAp24 antibodies. (b) Quantification of the percentage of VLP production of Gag and derivatives as described in Materials and Methods section (n= number of independent experiments). Gag and its derivatives used in this study are WT, NL4.3, WM and MACA-SP1. Values are mean±C.I. MW: molecular weight.

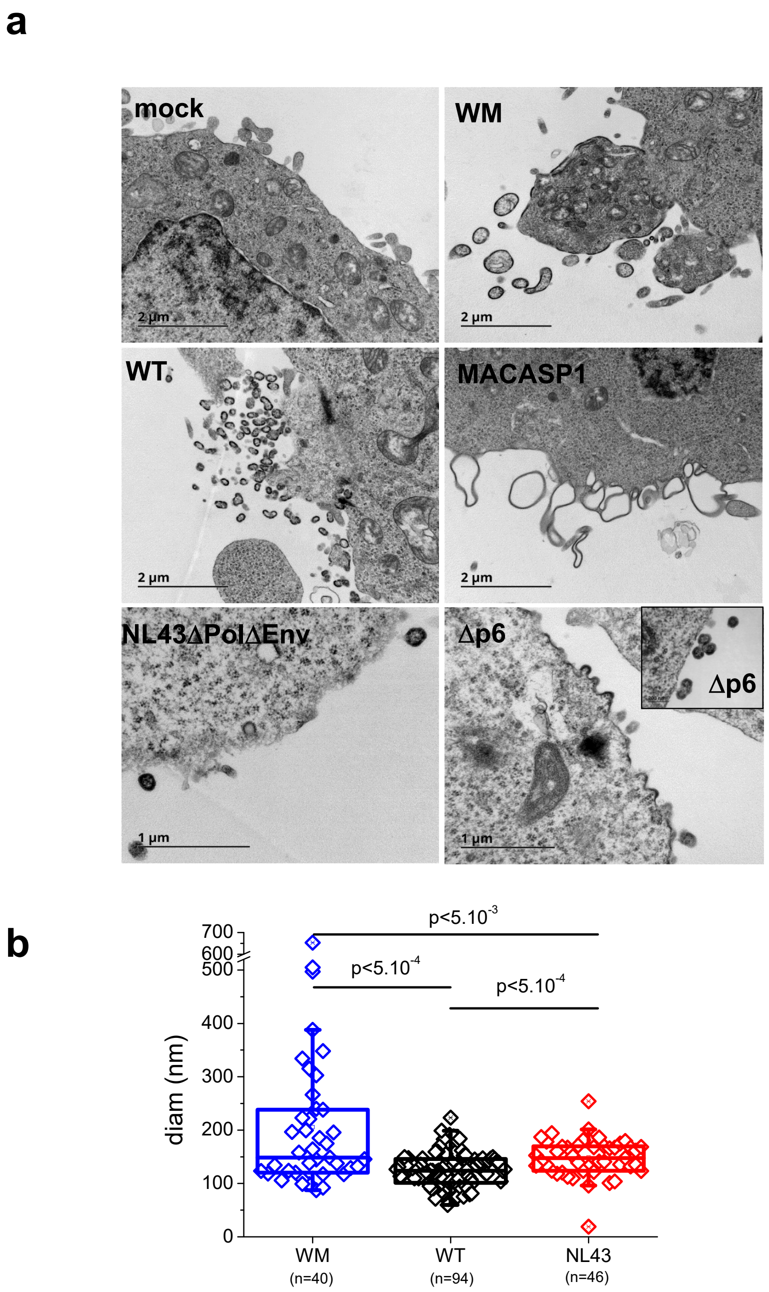

Fig. S2 Budding and VLP morphogenesis for Gag(i)mEOS2 and assembly mutants analysed by transmission electronic microscopy (TEM).(a) TEM images show typical VLP budding from HEK293T cells transfected with vector alone (mock), WT Gag, WM, MACA-SP1 and Δp6, or NL4.3ΔPolΔEnv (all internally tagged with mEOS2). (b) VLP diameter in cells transfected with Gag variants that could form VLPs (WT Gag, WM and NL4.3ΔPolΔEnvGag), p values are the results of Student's t tests.

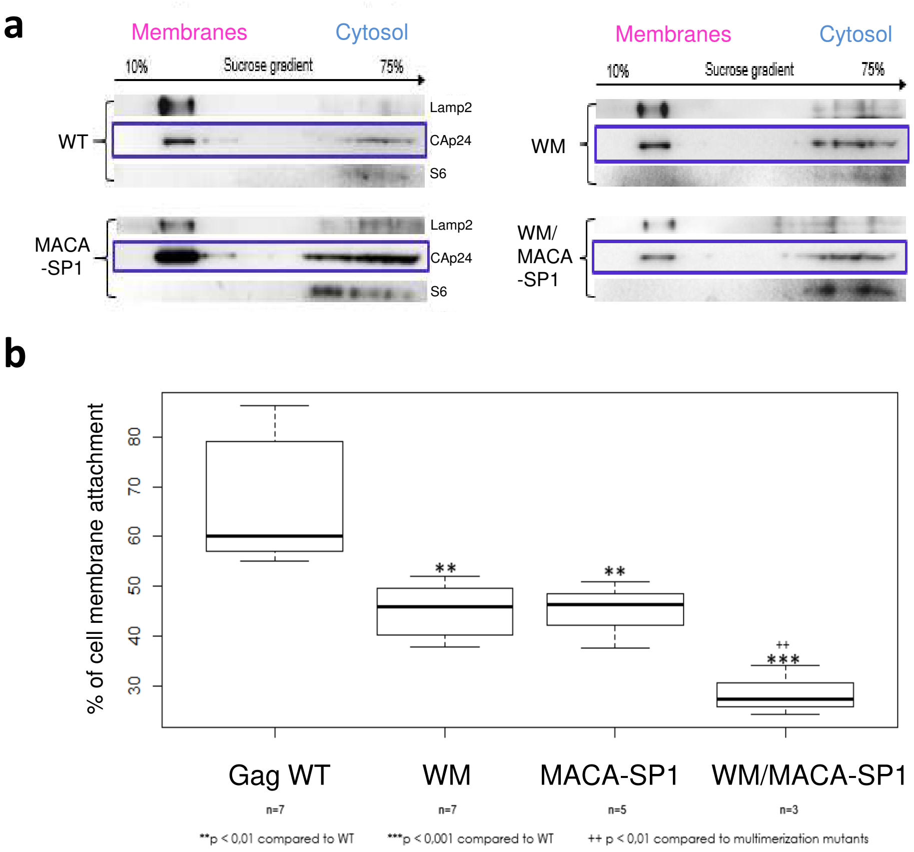

Fig. S3 Cell membrane attachment of Gag(i)mEOS2 (WT and assembly mutants).(a) Cell membrane flotation assays using post-nuclear supernatants from transfected HEK293T cells and detection of Gag proteins found in the cytosolic and membrane fractions by western blotting. (b) Quantitative analysis of Gag expression at the cell membrane (at least 6 independent experiments) showed that 60-80% of WT Gag was bound to cell membranes, and that this percentage decreased to 40% for the WM and MACA-SP1 mutants and below 30% for the WM/MACA-SP1 double mutant. $**p < 0.01$ and $***p < 0.001$ (Student t test, compared with WT Gag).

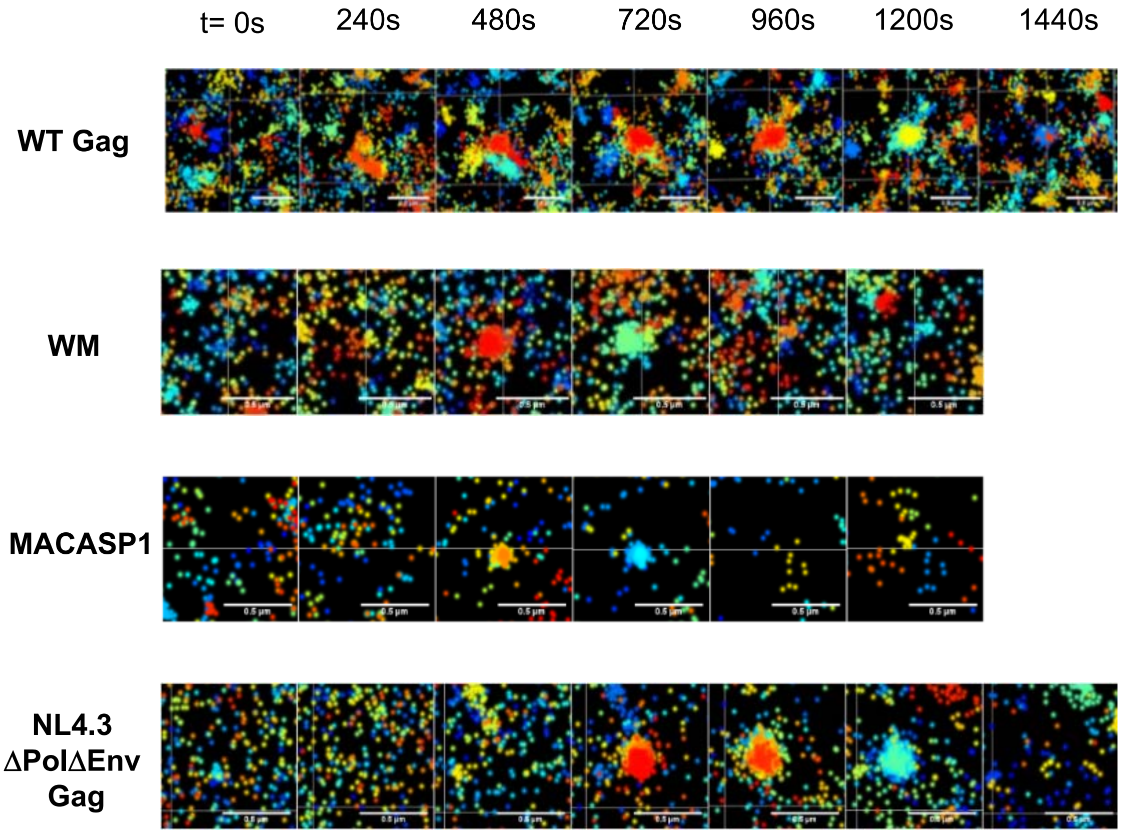

Fig. S4 Monitoring HIV-1 Gag(i)mEOS2 assembly by time-evolution of single molecule density in living T cells: Gag(i)mEOS2-expressing Jurkat T cells were seeded on poly-lysine-coated slides with microscope buffer containing 100nm TetraSpeck™ microspheres and live PALM/TIRF microscopy was performed at 37° C. Typical examples of density changes over time during assembly of WT Gag and mutants are depicted from top to bottom. Every image represents all the localizations observed during 4 minutes in and around identified assembly sites. These time pooled localisation are represented at increasing time of acquisition from left to right (0 to 1440 s).

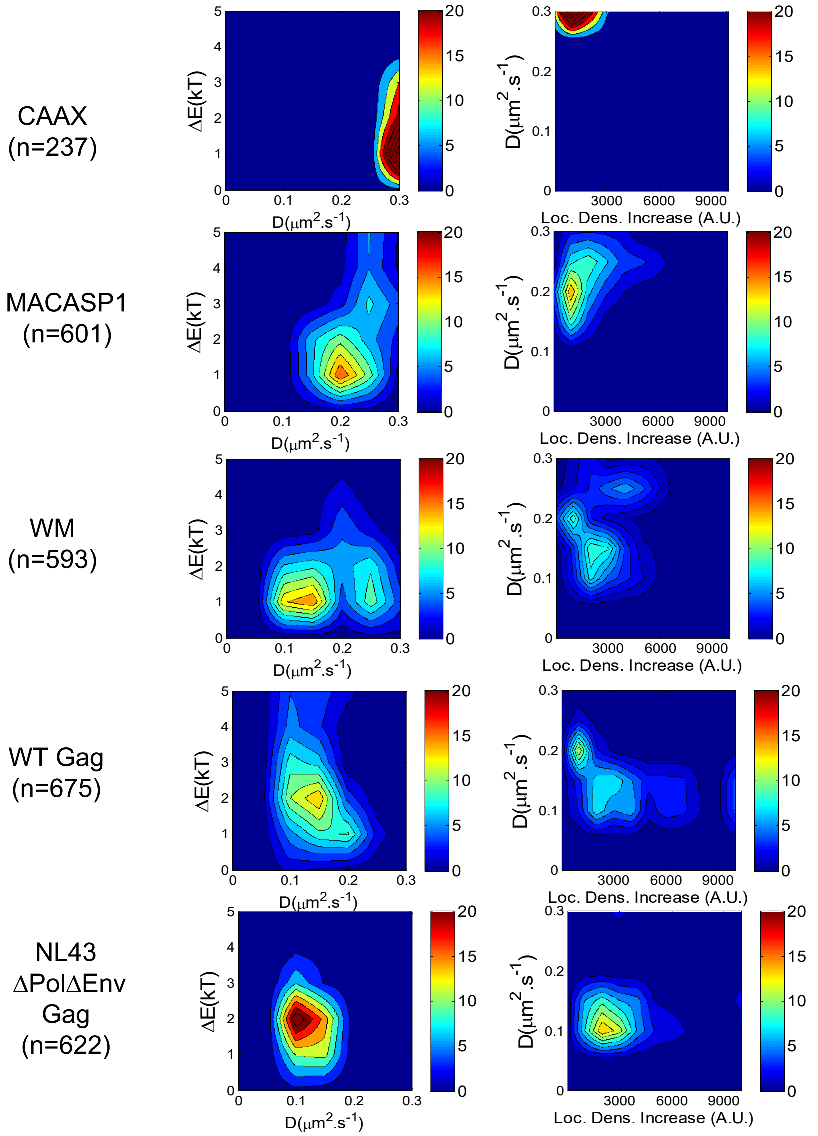

Fig. S5 **Normalized distribution diagrams of all identified assembling clusters.** Normalized distribution diagrams of the mean diffusion and the trapping energy (left), the mean diffusion and the localisation density increase (right) for each experimental condition, from CAAX(i)mEOS2 (no assembly) to WT Gag and NL4.3ΔPolΔEnv Gag (highest assembly efficiency).

S1 Video. Movie showing single-particle trajectories of HIV-1 WT Gag(i)mEOS2 (left) and their accumulation (right) during WT Gag assembly at the Jurkat T-cell plasma membrane. In the upper right part of the analysed surface, the formation of a particle can be observed (accumulation of trajectories in the right part of the movie) as well as the directed trajectories obtained during particle assembly (left part of the movie). Note the strong increase of the trajectory directionality during VLP assembly. In this movie, trajectories were reconstituted from the assignment graphs between images. The trajectories depicted in the movie are only for illustration. They were never used to reconstruct diffusivity and potential energy maps.

S2 Video. Movie showing the temporal changes of potential energy (left) and diffusivity (right) maps during HIV-1 WT Gag assembly and particle formation and release (in a selected area of the particle observed in video S1). This movie clearly shows that at the assembly site: i) the tessellation becomes progressively more narrow (indicating increased localization density) and is automatically reorganized, ii) the diffusivity decreases, and iii) the difference in energy with the outside increases with time during particle formation. These parameters go back to the initial values after particle release. Note the different temporal dynamics of the potential energy map when compared with the diffusion map and the differences in the spatial extent of the diffusion and energy maps.

S3 Video. Movie showing the temporal changes of potential energy maps (left) and the diffusivity (right) of an already assembled, but unreleased Δp6 mutant. From this movie, it can be clearly seen that the energy and diffusivity maps do not change over time for this mutant. One can still identify already formed particles by the narrowing of the tessellation.

Table S1. As illustrated in the main text, cluster diameters follow log-normal distributions. Therefore, two-independant sample Student t tests has been performed on the log values of the clusters diameters. This table contents the resulting p-values of this test. Difference in the mean values is considered to be significant for $p < 5.10^{-2}$

Gag variant	WT Gag	NL4.3ΔPolΔEnv Gag	WM	Δp6	VLP
WT Gag	1	—-	—-	—-	—-
NL4.3ΔPolΔEnv Gag	0.95	1	—-	—-	—-
WM	5.10^{-14}	5.10^{-13}	1	—-	—-
Δp6	5.10^{-6}	3.10^{-6}	0.16	1	—-
VLP	1.10^{-17}	2.10^{-17}	1.10^{-9}	1.10^{-4}	1

Table S2. As reported by Ivanchenko *et al.*[?] , HIV-1 particle formation can be divided in three steps:

- a density increasing phase that could correspond to Gag-Gag assembly,
- a plateau phase
- a particle-releasing phase.

In our curves showing the density increase over time (Fig.4d), these three phases could be observed. Because the third phase could be caused by different reasons, we decided to measure only the time length of the two first phases for the three Gag variants leading to particle assembly and release. Values are mean $\pm$ C.I.

Gag variant	Total number of VLPs	Duration of phase 1 Assembly	Duration of phase 2 Plateau
WM	36	360±64 s	376±55 s
WT Gag	76	396±48 s	355±34 s
NL4.3ΔPolΔEnv Gag	91	302±31 s	351±45 s

Table S3 This table contents the resulting p-values of a two independent samples Student's t test performed on the energy values measured in this work.

Gag variant	WT Gag	NL4.3ΔPolΔEnv Gag	WM
WT Gag	1	—-	—-
NL4.3ΔPolΔEnv Gag	0.92	1	—-
WM	3.10^{-6}	4.10^{-5}	1

Table S4 This table contents the resulting p-values of Kolmogorov-Smirnov test performed on the distribution of the difference in maximum density time and maximum energy time.

Gag variant	WT Gag	NL4.3ΔPolΔEnv Gag	WM
WT Gag	1	—-	—-
NL4.3ΔPolΔEnv Gag	$8.8.10^{-3}$	1	—-
WM	$1.9.10^{-3}$	$4.8.10^{-3}$	1

Detailed Materials and Methods

Buffers

The same microscopy buffer (MB) was used for fixed and live cell observations (150mM NaCl, 20mM HEPES pH7.4, 1mM CaCl2, 5mM KCl, 1mM MgCl2 pH 7-7.4). TNE buffer: 10mM Tris-HCl, 100mM NaCl, 1mM EDTA, pH 7.4. RIPA buffer: 150mM NaCl, 20mM Tris-HCl [pH 8], 1% NP-40, 0.1% SDS, 0.2mM EDTA, pH 8. Dulbecco's PBS was from Gibco.

Membrane flotation assay

For each condition, 6.10^6 HEK293T cells were transfected with the corresponding plasmids and harvested 24h post-transfection, lysed, and handled as described before[?]. Briefly, cells were washed with ice-cold PBS and resuspended in Tris-HCl containing 4mM EDTA and 1X Complete protease inhibitor cocktail (Roche). Cell suspensions were lysed using a Dounce homogenizer, and then centrifuged at 600g for 3min to obtain Post-Nuclear Supernatants (PNS). A cushion of 820µL of 75% (wt/vol) sucrose in TNE buffer (25mM Tris-HCl, 4mM EDTA, 150mM NaCl) was loaded at the bottom of an ultracentrifuge tube and mixed with 180 µL of PNS adjusted to 150mM NaCl. Two millilitres and 300 µL of 50% (wt/ml) sucrose cushion followed by 0.9 mL of 10% (wt/ml) sucrose cushion were then layered to obtain the gradient that was then centrifuged in a Beckmann SW60Ti rotor at 35,000 rpm, 4°C, overnight. Eight fractions were collected from the top to the bottom and analysed by western blotting. The percentage of membrane-bound Gag was calculated as in 2, by measuring the percentage of membrane-bound Gag protein in the PNS.

Transmission electron microscopy

Cells were fixed in 4% paraformaldehyde and 1% glutaraldehyde in 0.1M phosphate buffer (pH 7.2) for 48h, washed with PBS, post-fixed in 1% osmium tetroxide for 1h and dehydrated in a graded series of ethanol solutions. Cell pellets were embedded in EPON™ resin (Sigma) that was allowed to polymerize at 60°C for 48h. Ultrathin sections were cut, stained with 5% uranyl acetate and 5% lead citrate and deposited on collodion-coated grids for examination using a JEOL 1230 transmission electron microscope.

Detailled method of the Bayesian statistical analysis of single Gag analysis

As mentioned in the main text, the pipeline was organized in five steps:

- Single Molecule Localization
- Non-tracking with Graph Assignment
- Selection of the Regions of interest
- Time-Evolving Bayesian Inference Analysis
- Time-Evolving Feature Extraction from Inferred Maps

Single Molecule Localization

Single molecule localization was performed in MATLAB after implementation of the slightly modified MTT algorithm[?] to include it in a pipeline. These modifications did not affect the detection algorithm.

Non-tracking with Graph Assignment

To limit errors due to tracking algorithms[?], we did not track single molecules, but used optimal assignments between consecutive images to extract Gag displacements[?]. Indeed, experimentally, trajectories of Gag proteins were short (on average, 5 frames). Short trajectories can reduce the efficiency of the tracking algorithm and increase the rate of mislinking that can have deleterious effect on parameter estimation. Therefore, only displacements rather than trajectories were used to perform the analysis. The experimental maximal instantaneous density was $\rho \sim 1\mu m^{-2}$. Thus, between the most dense consecutive images, the mixing factor was $\frac{\sqrt{D.dt}}{\sqrt{1/\rho}} \sim 0.1\xi$ was the ratio of the distance covered by a particle over the average distance between particles. This suggested limited mixing between consecutive frames and justified the use of the optimal assignment as an estimator of particles displacement. The maximal variation of detectable particles between consecutive frames was 20%. This can lead to highly non-bipartite graphs of connections between particles. Hence, we relied on the Kuhn-Munkres scheme [KM] to find the optimal assignments, based on the minimization of the total distance between consecutive images. To speed up the algorithm and further limit long-distance mislinking, biomolecules separated by more than d=360 nm between consecutive frames were not linked in the graph. For displaying purposes (like in video **??**, for example), trajectories were regenerated from the assignments between images by directly following links between image datasets.

Selection of the Regions of interest (ROI)

To provide robustness in the analysis, parameters to automate treatment were directly extracted from the properties of the recording and not based on prior knowledge of the virion formation dynamics. ROI selection was based on localization densities. ROIs were selected as squared areas of 2 μm in length centred on the maximum density. The number of ROI per cell was limited to 30. In each ROI, the effective centre of a VLP, reff, was defined as the point of highest densities (ρ^{tot}_{max}) (cumulated on the 80,000 frames). The effective radius, R_{eff}, of a VLP was defined as the average distance between reff and the points of density equal to (ρ^{tot}_{max}/4). In the analysis, points within R_{eff} were considered to be in the VLP. Depending on the density of maturing VLPs, more than one VLP could be present in a single ROI. All VLPs inside such a region were analysed.

Time Evolving Bayesian Inference Analysis

The molecule motion was analysed using Bayesian inferences. The dynamics of individual Gag proteins were approximated by using the Overdamped Langevin Equation (OLE):

$$\frac{dr}{dt} = -D_t(r)\frac{\nabla V_t^{eff}(r)}{k_b T} + \sqrt{2D_t(r)} o_{HK}\xi(t) \tag{S.1}$$

where $D_t(r)$ was the space varying diffusivity, $V_t^{eff}(r)$ the effective interaction potential, $\xi(t)$ a zero-averaged Gaussian noise and the stochastic integral (linked to the symbol o_{HK}) was interpreted according to Hänggi-Klimontovich. Written using an Itô interpretation, the OLE reads as

$$\frac{dr}{dt} = -D_t(r)(\frac{\nabla V_t^{eff}(r)}{k_b T} - \frac{\nabla D_t(r)}{D_t(r)}) + \sqrt{2D_t(r)}\xi(t) \tag{S.2}$$

where the spurious force $\nabla D_t(r)$ appears explicitly. We defined $V_t^{HK}(r) = V_t^{eff}(r) - k_B T log(D(r))$. $(D_t(r), V_t^{eff}(r))$ was considered to describe the statistical features that encode the effective dynamical characteristics of the environment concerning individual Gag dynamics. It describes the effective medium properties (approximated according to the OLE) experimented by a Gag protein during its recording time.

Bayesian Inference was used to extract $(D_t(r), V_t^{eff}(r))$from the assignment between images[?,?,?,?,?]. In (**??**) and (**??**), the index t shows that diffusion and potential fields can evolve with time, but at a time scale larger than the particle dynamics.

In a nutshell Bayesian inference features two steps: the derivation of the posterior probability distribution of the model parameters and sampling from the posterior distribution to estimate the parameters. Bayes formula states:

$$P(\{U\} \mid \{T\}) = \frac{P(\{T\} \mid \{U\})\,\pi(\{U\})}{P(\{T\})} \tag{S.3}$$

where $\{T\}$ is the set of experimental observations and $\{U\}$ is the set of model parameters (to be evaluated). In standard terminology, $P(\{U\} \mid \{T\})$ is the *posterior* distribution, $P(\{T\} \mid \{U\})$ is the *likelihood*, $P(\{U\})$ is the *prior* distribution, and $P(\{T\})$ is the *evidence* of the model. Here, the likelihood embodies the physical model and hypotheses regarding the acquisition of data. Prior distribution can represent either knowledge on the parameters before any measurements as well as can be used to regularise the inferred parameters as discussed below (this will be the case in this study).

Based on the OLE the The likelihood reads

$$P\left(\{d\mathbf{r_k}\}_{\mathbf{k}\in\mathscr{T}} \middle| \left(\mathbf{D}(\mathbf{r}), \mathbf{V^{HK}}(\mathbf{r})\right)\right) \propto \prod_{(l\in\mathscr{M})} \left(\prod_{k\in\mathscr{T}_l} \frac{\exp\left(-\frac{\left(d\mathbf{r}_\mathbf{k}^{\mu} - \mathbf{D_l}\nabla\mathbf{V_l^{HK}}\blacksquare\mathbf{t}/\mathbf{k_B T}\right)^2}{4\left(D_l + \frac{\sigma^2}{\Delta t}\right)\Delta t}\right)}{\left(4\pi\left(D_l + \frac{\sigma^2}{\Delta t}\right)\Delta t\right)} \right) \tag{S.4}$$

where $\mathscr{M}$ is the mesh, S_l is a subdomain in the mesh, D_l the value of the diffusivity $D(\mathbf{r})$ in the mesh subdomain l, V_l^{HK} the value of the potential $V_t^{HK}(\mathbf{r})$ in the mesh subdomain l, $\mathscr{T}$ is the total set of displacements, $\mathscr{T}_l$ is the subset of displacements happening in the mesh subdomain S_l, σ is the positioning noise modelled as a Gaussian process and Δt is the duration between two frames.

The Bayesian Inference approach provides for each maps the posterior distribution of the full set of parameters. Here, we use the Maximum A Posterior (MAP) as an estimator of the two maps $\left(D_t(\mathbf{r}), V_t^{eff}(\mathbf{r})\right)$. $V^{eff}(\mathbf{r})$ was directly deduced as $V^{eff}(\mathbf{r}) = V^{HK}(\mathbf{r}) + k_B T \log(D(\mathbf{r}))$. The time-evolution of the maps was directly the evolution of the MAP of each individual maps. Note that the OLE was the only model used to analyse the experimental set. As different models were not tested, the evaluation of the evidences for the chosen model will not be discussed.

Field regularizing priors were used:

$$\pi\left(D(\mathbf{r}), V^{HK}(\mathbf{r})\right) \propto \exp\left(-\alpha \int d^2\mathbf{r}\,(\nabla\mathbf{D}(\mathbf{r}))^2 - \delta \int \mathbf{d^2 r}\left(\nabla\mathbf{V^{HK}}(\mathbf{r})\right)^{\mathbf{2}}\right) \tag{S.5}$$

with $\alpha = 0.25\ \mu m^{-4} s^2$ and $\delta = 0.1\,(k_B T)^{-2}$. The choice of significant regularisation was motivated by the large variability of maps to be inferred. Maturing VLPs have densities varying largely with time. Yet, the analysis performed on the maps should not largely vary with the number of points during the time window. These regularising coefficients ensured that for all time windows no maps would exhibit abnormal features induced by the reduced number of points.

Time-Evolving Feature Extraction from Inferred Maps

The changing dynamics during VLP assembly led to high variability in particle density. The tessellation procedure described in[?] was modified to ensure more homogeneity in the structure of the spatial tessellation. The initial mesh was defined using unsupervised learning (k-mean). The average number of points per mesh was initialized to 40 points per mesh subdomains. This mesh was then corrected by reassigning all points belonging to subdomains with less than 10 detections. This procedure was done iteratively by removing only one subdomain per iteration. The final mesh was the Voronoi tessellation of the remaining centre of subdomains. This correction procedure had an effect mostly on areas outside the VLP. It ensured that measures could be done inside and outside the VLP. The time evolution of the VLP dynamics was assessed by time windowing. The window duration was set to 240s with a sliding time of 10s. This duration is a trade-off between ensuring a minimal number of points to perform reliable inference and ensuring a minimal progress in VLP maturation. The inference was performed independently on each time window. Independence allowed the map to be inferred independently of previous or future detections. It also avoided introducing bias by coupling the inference on multiple time windows. Hence, a typical time evolution of a VLP maturation lead to 136 maps of $(D_t(r), V_t^{eff}(r))$. In order to quantify different dynamics, several features were extracted from the time evolving maps.

Parameters

The effective centre of a VLP, r_{eff} was defined as the point of highest density ρ_{max}^{tot} (cumulated on the 80,000 frames). The effective radius, R_{eff}, of a VLP was defined as the average distance between r_{eff} and the points of density equal to $\frac{\rho_{max}^{tot}}{4}$. In the analysis, points within R_{eff} from r_{eff} were considered to be in the VLP. $I(j)$ was the set of mesh subdomains in the VLP at the time window j. Neighbours to a VLP or a predefined radius, R, were defined as the set of mesh subdomains in contact with the VLP or the circle of radius R. $M(j)$ was the set of neighbours at the time window j. $A(j)$ was the set of all subdomains of the mesh at the time window j.

Time-evolving density The VLP density inside a map (corresponding to a time window) was evaluated as $\rho(t) = \frac{N(t)}{(\pi R_{eff}^2)}$ where $N(t)$ was the number of localization in the VLP in the time window. The temporal evolution of the density for a VLP was directly computed as the density measure on the set of maps associated with that VLP.

Time-evolving diffusivity Three estimators were extracted from the diffusivity maps. The diffusivity in the VLP at the time window j was defined as $D_{in}(j) =< D >_I (j)$ where $< . >$ was the spatial average. The diffusivity around the VLP at the time window j was defined as $D_{out}(j) =< D >_M (j)$. The relative variation of diffusivity between outside and inside the VLP at the time window (j) was defined as $(j) = (D_{out}(j) - D_{in}(j))/(D_{out}(j))$.

Time-evolving effective trapping energy Three estimators were directly extracted from the potential maps. The depth of the VLP at the time window j was defined as $V^{eff}(t) =< V^{eff} >_{M_{2R_{eff}(j)}} - min_{I(j)} V^{eff}$.

Finally, for each VLP present in the analysed ROIs (i.e., 600 VLPs per mutant), the time-evolving density, diffusivity and potential were smoothed with a 10th order Savitsky-Golay filter to extract the following parameters:

- Localization Density Increase (LDI):

$$LDI = \rho_{max} - \rho_{min} \quad \text{(S.6)}$$

- Mean diffusivity in the assembly platform :

$$D = < D_{in} >_t \tag{S.7}$$

where $< . >$ was the time average.

- Maximum Trapping Energy :

$$\Delta E = \delta V^{eff}_{max} - \delta V^{eff}_{min} \tag{S.8}$$

The assembly time length was defined as the $1/e^2$ width of the Gaussian fit of the LDI peak. As the analysis led to $\backsimeq$ 100,000 maps of $(D_t(r), V^{eff}_t(r))$, the $(D_t(r), V^{eff}_t(r))$ values observed in each VLP were distribute in classes. Each class was renormalized to the total VLP number. This allowed generating $(V^{eff} = f(D), D = f(LDI))$ diagrams for each mutant.

The analysis in numbers: Big Data with single molecules

As for many scientific fields, single-molecule science is entering the Big Data Age. Although, single molecule image datasets remain of reasonable size (70 Go for this study), the amount of output files and results are very significant. This analysis lead to 100,000 maps of $(D_t(r), V^{eff}_t(r))$. Then, features of these maps were analysed, generating in total, including the intermediary files, 190 Go of results. We can see the change of scale between the number of cells tested and the size of results. Large amounts of information about biological dynamics are stored in the random walks of biomolecules. Increasing the number of analysed cells will lead to a massive amount of result files and will make the analysis of the diversity of processes even more complex. Here, a rather direct analysis, based on effective physical maps of the cellular environment, showed directly identifiable differences in dynamics of Gag variants. Increasing the number of cells will increase the dynamics diversity and will require more sophisticated tools to statistically characterize the effects at the population scale[?]. The ever-increasing amount of data and the necessity of multi-platform analysis are currently addressed with the development of a new Inference Pipeline in Python and a new compressed file format to store all analyses performed on single-molecule dynamics.

References

1. Ivanchenko, S. *et al.* Dynamics of HIV-1 assembly and release. *PLoS pathogens* **5**, e1000652–e1000652 (2009). DOI 10.1371/journal.ppat.1000652.

2. Kerviel, A. *et al.* Involvement of an Arginine Triplet in M1 Matrix Protein Interaction with Membranes and in M1 Recruitment into Virus-Like Particles of the Influenza A(H1n1)pdm09 Virus. *PloS One* **11**, e0165421 (2016). DOI 10.1371/journal.pone.0165421.

3. Sergé, A., Bertaux, N., Rigneault, H. & Marguet, D. Dynamic multiple-target tracing to probe spatiotemporal cartography of cell membranes. *Nat. Methods* **5**, 687–694 (2008). DOI 10.1038/nmeth.1233.

4. Chenouard, N. *et al.* Objective comparison of particle tracking methods. *Nat. Methods* **11**, 281–289 (2014). DOI 10.1038/nmeth.2808.

5. Chertkov, M., Kroc, L., Krzakala, F., Vergassola, M. & Zdeborová, L. Inference in particle tracking experiments by passing messages between images. *Proc. Natl. Acad. Sci. United States Am.* **107**, 7663–7668 (2010). DOI 10.1073/pnas.0910994107.

6. Harney, H. L. Form Invariance II: Natural x. In *Bayesian Inference*, 95–108 (Springer Berlin Heidelberg, Berlin, Heidelberg, 2003). DOI: 10.1007/978-3-662-06006-3_11.

7. Chang, J. C., Fok, P.-W. & Chou, T. Bayesian Uncertainty Quantification for Bond Energies and Mobilities Using Path Integral Analysis. *Biophys. J.* **109**, 966–974 (2015). DOI 10.1016/j.bpj.2015.07.028.

8. El Beheiry, M., Dahan, M. & Masson, J.-B. InferenceMAP: mapping of single-molecule dynamics with Bayesian inference. *Nat. methods* **12**, 594–595 (2015). DOI 10.1038/nmeth.3441.

9. van de Meent, J.-W., Bronson, J. E., Wiggins, C. H. & Gonzalez, R. L. Empirical Bayes methods enable advanced population-level analyses of single-molecule FRET experiments. *Biophys. J.* **106**, 1327–1337 (2014). DOI 10.1016/j.bpj.2013.12.055.

10. Persson, F., Lindén, M., Unoson, C. & Elf, J. Extracting intracellular diffusive states and transition rates from single-molecule tracking data. *Nat. Methods* **10**, 265–269 (2013). DOI 10.1038/nmeth.2367.

11. Bishop, C. M. *Pattern recognition and machine learning*. Information science and statistics (Springer, New York, NY, 2009), corrected at 8. printing 2009 edn. OCLC: 845772798.
